# Marketing communication strategies on labels of food products consumed by children

**DOI:** 10.11606/s1518-8787.2023057004614

**Published:** 2023-11-17

**Authors:** Luciana Azevedo Maldonado, Silvia Cristina Farias, Kelly Veloso da Cruz, Bruna Pereira dos Santos, Luciana Maria Cerqueira Castro, Inês Rugani Ribeiro de Castro

**Affiliations:** I Universidade do Estado do Rio de Janeiro Instituto de Nutrição Departamento de Nutrição Social Rio de Janeiro RJ Brasil Universidade do Estado do Rio de Janeiro. Instituto de Nutrição. Departamento de Nutrição Social. Rio de Janeiro, RJ, Brasil.; II Universidade do Estado do Rio de Janeiro Rio de Janeiro RJ Brasil Universidade do Estado do Rio de Janeiro. Programa de Pós-graduação em Alimentação, Nutrição e Saúde. Rio de Janeiro, RJ, Brasil.; III Universidade do Estado do Rio de Janeiro Instituto de Nutrição Rio de Janeiro RJ Brasil Universidade do Estado do Rio de Janeiro. Instituto de Nutrição. Rio de Janeiro, RJ, Brasil.

**Keywords:** Children's Health, Food Labeling, Food Advertising, Child Nutrition

## Abstract

**OBJECTIVE:**

Analyze marketing communication strategies (MCS) of labels of food products consumed by children under 5 years of age from the Brazilian National Health System (SUS) in the city of Rio de Janeiro.

**METHODS:**

In total, 390 labels of ultra-processed foods and industrialized baby foods were analyzed. The products were organized by similarity into 24 groups. Photographs of labels from each group were analyzed to identify the MCS, which were categorized into “presence of characters and/or celebrities,” “emotional appeal,” “freebies offering,” “health appeal,” “sensory stimulation,” “brand or slogan use,” “promotional price,” “advertisement under advertisement,” and “sustainability appeal.” The percentage frequency of labels according to the number of MCS per label; the total and average frequency of MCS according to the food group; the frequency of MCS type according to the food group; and communication resources by type of MCS were computed.

**RESULTS:**

1 to 19 strategies were found per label and an average of 7.2 MCS per label, totaling 2,792 occurrences. The MCS “sensory stimulation,” “health appeal,” “brand or slogan use,” and “advertisement under advertising” were observed in all food groups. “Freebies offering” and “promotional price” were observed in eight and six food groups, respectively. In food groups of bread; dairy products; and sweets, candies, and goodies, all nine types of MCS included in the study were identified. The groups that presented fewer types of MCS (n=5) were: peanuts, instant noodles, and margarines. Of the total MCS identified on the labels, the most frequent were “sensory stimulation” (29.4%) and “health appeal” (18.2%); and the least frequent were “freebies offering” (0.8%) and “promotional price” (0.4%). The “emotional appeal” strategy presented the highest diversity of communication resources.

**CONCLUSION:**

Rigorous regulatory measures are required to protect consumers from massive exposure to MCS on food labels.

## INTRODUCTION

Excess weight in children has significantly increased in the last decades, affecting 38.2 million children under 5 years of age worldwide^1,2^. An important factor for this phenomenon is the increase in consumption of ultra-processed foods (UPF). In Brazil, in 2019, the prevalence of UPF consumption among children aged 6 to 23 and 24 to 59 months was 80.5% and 93%, respectively^3^.

UPFs are hyper-palatable foods that compromise healthy eating habits. Most of them have an unbalanced nutritional composition, with high amounts of fats, sugars, salt, and food additives with cosmetic roles, such as emulsifiers, thickeners, coloring materials, sweeteners, among others, not used in homemade preparations^4^. In addition to being associated with poor diet quality and obesity, the consumption of these foods is also related to other unfavorable health outcomes, including tooth decay, increased risk of cardiovascular disease, insulin resistance, type 2 diabetes, and micronutrient deficiency^5,6^.

The exposure of children to these products is a consequence of an increase in working hours of parents, including the time spent commuting between home and work, the overload of women with household chores, low cooking skills, lack of healthy options in schools and other food environments^7–9^, and the massive use of abusive and misleading marketing strategies for these products, including those directed to children^10^.

These strategies include the use of packaging, given its strategic role in the communication between the manufacturer and the consumer^11^. Packaging attracts consumers and, when choosing food, establishes a direct communication channel for the supposed advantages of the product. Food industries have invested in the development of packaging design, constituting a powerful communication channel between the manufacturer and the consumer that has been increasingly used to spread meanings and images that promote the acceptance, repurchase, and use of products^11^.

Marketing strategies focus their actions on children due to their great ability to persuade their parents^7^. Exposure to advertisements and other marketing communication strategies (MCS) to promote UPFs broadcasted in the most diverse media channels – mainly television and the internet – are more harmful to children and adolescents, due to the lack of cognitive maturity for discernment and understanding of advertising and hidden objectives^12^. For this reason, food advertisements worldwide for children and adolescents have been addressed in public policies, such as those that regulate food advertising and labeling^1,13^.

Studies on this topic have analyzed specific foods or food groups available in supermarkets or based on some theoretical criteria^14^. This study seeks to contribute to the state-of-the-art knowledge of food advertising and labeling using a different approach: the analysis of the main MCS present on labels of UPFs and industrialized baby foods that are actually consumed by children under 5 years of age from the Brazilian National Health System (SUS) in the city of Rio de Janeiro.

## METHODS

This study was based on a survey carried out in 2014 with a probabilistic sample of 536 children aged 6 to 59 months, assisted in the basic health services in the city of Rio de Janeiro. Trained nutritionists applied 24-hour dietary recalls (24HR) to parents or guardians of children. Information was collected regarding the types of foods, quantities, preparation methods, time and place of consumption, and, in the case of processed foods and UPFs, their respective brands and flavors. A detailed description of this investigation is available in Anastácio et al.^15^

After analyzing all foods consumed, UPFs were identified with the NOVA food classification. Industrialized baby foods were also included as they present similar attributes to those of UPFs^16^. In case of doubt, the authors of the NOVA food classification were contacted. Then, 459 products were selected for the study.

Between March and December 2015, the labels of UPFs and industrialized baby foods, according to the flavor and/or brand mentioned in the 24HR, were photographed in stores located in different areas of the city. All types of packaging with the same product available in these stores were photographed (e.g. soft drinks in cans and PET bottles). In this case, only one label per product was included in an image bank – the label presenting the highest number of MCS.

Before going to the stores, field researchers were trained to photograph all faces of the labels in order to obtain the necessary information for all study objectives. The training included a presentation class and a practical activity in stores. The photos were taken with a cell phone and stored in Google Drive files.

In total, 390 labels of UPFs and industrialized baby foods were analyzed; 69 (15%) labels out of total 459 could not be analyzed for the following reasons: photo available from only one face of the label that contained the nutritional table or absence of the label photo (product not found in stores or on the internet). In general, the image bank had three to four photos of the various faces of every product. However, 79 products (20.3% of 390 analyzed products) did not have photos of all label faces.

The products were organized by similarity into 24 food groups, described in Chart 1.

**Chart 1:** Description of products analyzed by food groups consumed by children under 5 years of age from the Brazilian National Health System (SUS). Rio de Janeiro, Brazil, 2014.

**Products analyzed by food groups**
**Chocolate powder and similar products**
Chocolate powder in plastic jar: Showcau® (400g), Toddy® (200g, 400g, 800g)
Chocolate powder in aluminum can: Nescau® (400g)
Chocolate powder in plastic sachet: Mágico® (200g and 400g)
Ovomaltine chocolate powder with crunchy flakes in plastic sachet: Ovomaltine® (300g)
Strawberry flavored powder in aluminum can: Nesquik® (380g)
**Peanuts**
Peanuts in laminated plastic packaging: Mendorato (500g)
**Soy drinks**
Drinks in carton packaging: Ades® (pineapple, orange, apple, strawberry, peach, soy, grape 1000ml, peach 200ml), Nestlé® (Sollys apple 1000ml and orange 200ml), Shefa® (original and grape 1000ml), Sufresh® (apple 1000ml)
**Cookies**
Cookie in carton packaging: BelVita® (honey and cocoa 30g)
Cornstarch cookie in plastic packaging: Duchen® (200g), Mabel® (400g), Marilan® (400g), Piraquê® (cornstarch and Marie type 200g), Vitarella® (traditional and milk type 400g)
Sandwich cookie in laminated plastic packaging: Adria® (chocolate tart, white chocolate, milk chocolate, Swiss chocolate, strawberry and chocolate, semi-sweet chocolate 150g, strawberry 160g, Plugados chocolate and lemon 150g), Bauducco (chocolate 130g and Gulosos 140g), Mabel® (chocolate 140g), Marilan® (strawberry 160g), Nestlé® (Bono chocolate 140g, Passatempo chocolate 140g, strawberry 20g and 140g), Parati® (chocolate 115g, chocolate 140g), Piraquê® (pineapple, chocolate, lemon, strawberry 200g), Richester® (Amori chocolate 140g), Trakinas® (chocolate and strawberry 143g, strawberry and chocolate 136g)
Guava sandwich cookie in laminated plastic packaging: Bauducco® (140g) and Piraquê® (100g)
Donut-shaped cookie in laminated plastic packaging: Mabel® (banana and cinnamon, coconut, milk and cream 400g), Piraquê® (milk 400g)
Cookie without filling in laminated plastic packaging: Nestlé® (Passatempo milk 150g)
*Sequilhos*, dry cornstarch cookie in laminated plastic packaging: Vovó Delma® (milk 300g)
Drop cookies in laminated plastic packaging: Bauducco® (chocolate (110g)
Malted milk cookie in laminated plastic packaging: Piraquê® (crunchy and natural chocolate 200g)
Wafer cookie in laminated plastic packaging: Bauducco® (chocolate 130g), Mirabel® (chocolate and strawberry 40g), Piraquê® (chocolate 160g)
**Crackers and snacks**
Salt & water crackers in laminated plastic packaging: Adria® (200g), Richester® (200g), Vitarella® (400g)
Crackers and snacks in laminated plastic packaging: Adria® (cream cracker 200g), Bauducco® (light cracker 200g), Club Social® (bacon 156g, whole wheat 288g, original 155g, pizza 141g), Elma Chips® (Baconzitos 55g, Cheetos parmesan 45g, cheese 59g, creamy cheese 61g, Fandangos cheese and ham 175g, Ruffles original 24g and 100g), Fofura® (onion, barbecue, cheese 100g), Mabel® (cracker 200g), Marilan® (cracker 400g), Pit Stop cheese and original 162g), Nestlé® (cream cracker: whole wheat 170g, sesame seed 160g, Nesfit 126g), Piraquê® (cracker 200g, whole wheat cracker 240g, cheese flavored 100g, ham flavored 100g, savory 100g), Richester® (cracker 200g, sesame seed cream cracker 200g), Torcida® (barbecue 50g, cheese 80g, pizza 80g), Triunfo® (cracker 200g), Vitarella® (cracker and cracker crocks 400g, Saltvip 156g)
Potato chips in laminated plastic packaging: Elma Chips® (Sensações 45g)
Cassava starch cookie in plastic packaging: BC® (100g), Vale D'ouro® (100g)
**Ready-made cakes**
Cake in plastic sachet: Ana Maria® (carrot and chocolate 40g and 80g; vanilla with chocolate drops and chocolate filling 80g)
Cake in plastic sachet: Bauducco® (chocolate cream 40g; orange 220g)
Strawberry cake in plastic sachet: Panco® (70g)
Carrot cake mix in plastic sachet: Santa Amália® (400g)
Chocolate cake mix in plastic sachet: Dona Benta® (400g)
**Breakfast cereals**
Breakfast cereals in carton box: Alca Foods® (300g), Nestlé® (270g and 330g), Kelloggs® (300g)
**Sweets, candies, and goodies**
Candies in plastic bags: Arcor® (7 Belo 150g), Butter Toffees® (milk 750g), Dori® (yogurt 600g), Embaré® (traditional milk 150g and condensed milk 840g), Fini® (strawberry 86g), Freegells® (sour strawberry 420g), Gamadinho® (peanut sweet 600g), Juquinha® (tutti-frutti 300g), Peccin® (strawberry, yogurt, orange 600g)
Candies in carton box: Freegells® (tangerine 31.7g), Mentos® (fruit 428.8g and ice mint 114g), Trident® (mint 32g)
Chocolate candies in plastic bag: Garoto® (Serenata de Amor 19g)
Gum in carton box: Adams® (tutti-frutti 280g)
Chocolate in carton box: Lacta® (Bis chocolate 126g), Garoto® (Batom milk chocolate 480g), Nestlé® (Alpino chocolate 450g), Marisbel (chocolate 300g)
Chocolate in aluminum bag: Kinder Ovo® (chocolate 20g)
Chocolate in aluminum can: Nestlé® (Moça doceria de brigadeiro 385g)
Chocolate in plastic bag: Arcor® (Tortuguita chocolate with cereal 140g), Cacau show® (white chocolate 20g), Dr. Oetker® (soft sprinkles 130g), Garoto® (white chocolate 150g), Lacta® (Diamante Negro 150g), M&M® (peanut and chocolate 52g), Nestlé® (Classic milk chocolate and semi-sweet chocolate 150g)
Hazelnut cream: Nucita® (chocolate and hazelnut)
Dulce de leche in plastic jar: Pingo de Leite® (500g)
Gelatin in cardton box: Dr. Oetker® (strawberry 30g), Lual® (raspberry 30g), Royal® (raspberry, lemon, strawberry 35g), Sol® (lemon, strawberry, grape 35g)
Mocotó jelly in a glass jar: Arisco® (strawberry, natural, tutti-frutti 220g), Ducopo® (strawberry and natural 180g)
Glucose syrup in plastic jar: Karo® (350g)
Condensed milk in aluminum can: Nestlé® (Leite Moça 395g)
Lollipop in plastic bag: Peccin® (tutti-frutti 672g), Simas® (Pop cherry 750g)
Ice cream: Bob's® (vanilla cone with dulce de leche syrup), Kibom® (Chicabon, Creme, and Cremosíssimo Napolitano), McDonald's® (vanilla cone with chocolate syrup)
**Flours**
Flours in aluminum cans for flavored porridges: Nestlé® (Neston instant vitamin, strawberry, pear, banana, cereal, and papaya, apple, banana, cereal; and Mucilon corn 400g)
Flours for flavored porridges in plastic sachets: Nestlé® (Neston instant vitamin - banana, papaya, apple, cereal, and 3 cereal flakes 210g, and Mucilon rice, rice and oat, and multi cereals (230g), Nutri Day® (rice 200g), Nutrilon® (oat 230g), Vitalon® (6 cereals 200g)
Dairy flour in plastic sachet: Nestlé® (210g)
Flour for rice cream flavored porridge with vitamins and minerals in plastic bag: Chinezinho® (200g), Yoki® (200g)
Flour for cornstarch-based porridge in a carton box: Cremogema Maizena® (traditional, strawberry, banana, chocolate 200g)
**Ready-made toasted manioc flour**
Ready-made toasted manioc flour in a laminated bag: Sinhá® (seasoned bacon and seasoned cassava 250g), Yoki® (seasoned cassava 500g)
**Dairy products**
Flavored dairy drink in carton: Elegê® (strawberry 200ml), Del Valle® (chocolate 200ml), Nestlé® (Mucilon Prontinho orange, apple and pear, and banana and peach 190ml), Toddynho® (chocolate 200ml)
Flavored dairy drink in plastic bottle: Bialini® (strawberry 800g), Danone® (strawberry 170g and coconut 900g), Elegê® (strawberry 900g), Itambé® (strawberry 600g), Nestlé® (apple and banana 170g), Vigor® (strawberry 180g), Yofruta® (strawberry 170g)
Cream cheese in plastic jar: Philadelphia® (150g)
Flavored yogurts in plastic trays: Batavo® (Grego strawberry 400g), Danone® (Danoninho strawberry and banana, strawberry and green apple 360g, Danoninho strawberry and banana 540g), Elegê® (petit-suisse strawberry (Bob Esponja) 320g), Itambé® (petit-suisse strawberry and strawberry and banana 360g), Nestlé® (Chambinho strawberry 320g, Ninho strawberry and three flavors 600g and strawberry 540g), Paulista® (petit-suisse strawberry (Galinha Pintadinha) 330g), Vigor® (chocolate 200g, Grego Kids strawberry 360g, strawberry and strawberry and fruits 540g)
Flavored yogurts in plastic jars: Frimesa® (strawberry 165g), Paulista® (Grego with strawberry syrup 95g) and Vigor® (Grego vanilla with strawberry syrup 95g, Grego traditional and vanilla 100g, and orange, carrot and honey 170g)
Fermented milk in carton box: Elegê® (80ml)
Fermented milk in plastic bottle: Actimel® (600g), Batavo® (480g), Danone® (450g and 600g), Nestlé® (450g), Paulista® (450g), Yakult® (480g)
Polenguinho® in carton box: (with 8 units of 17g each)
Creamy cheese in a glass cup: Itambé® (220g)
Creamy cheese in a plastic cup: Godam® (light 200g), Polenghi® (regular and light 200g), Sadia® (200g), Vigor® (200g)
**Instant noodles**
Instant noodles in plastic bag: Maggi® (beef and chicken 85g), Nissin® (free-range chicken and chicken 85g), Richester® (beef 85g), Vigor® (beef 85g)
**Margarines**
Margarine in plastic jar: Delícia® (500g), Doriana® (250g and 500g) and Qualy® (500g)
**Breads**
Burger bun in plastic bag: Plus Vita® (480g)
Hot dog bun in plastic bag: Panco® (300g), Plus Vita® (360g), Wickbold® (300g)
Sliced bread in plastic bag: Plus Vita® (500g)
Toasted bread in plastic bag: Bauducco® (160g), Marilan® (150g), Wickbold® (140g)
**Industrialized baby foods**
Industrialized baby foods in glass cups: Nestlé® (fruits 120g, and rice, beans and beef 170g)
**Microwave popcorn**
Microwave popcorn in plastic sachet: Yoki® (caramel flavor 160g, butter flavor 100g, and natural flavor 100g)
**Reconstituted meat products**
Burger in a carton box: Perdigão® (chicken and beef 672g), Sadia® (beef 672g)
Calabrian sausage in plastic bag: Perdigão® (400g) and Seara® (400g)
Mortadella in plastic bag: Perdigão® (1000g)
Nuggets in carton box: Sadia® (traditional 300g), Seara® (traditional Turma da Mônica 300g)
Ham in plastic bag: Sadia® (200g)
Hot dog sausage in plastic bag: Perdigão® (500g) and Sadia® (500g)
**Soft drinks**
Soft drink in plastic bottle: Coca-Cola® (2000ml), Dolly® (guarana 350ml), Fanta® (orange and grape 2000ml), Grapette® (raspberry 290ml), Pepsi® (2500ml), Schin® (guarana 2000ml), Sprite® (lemon 2000ml), Tobi® (cola and guarana 350ml)
Guarana drinks
Drink in plastic cup: Açaicamp® (açaí 285ml), Ativ plus® (guarana 290ml), Guaracamp® (guarana 285ml), Guaraplus® (guarana 290ml), Guaravita® (guarana 290ml)
**Juice cartons**
Juice in carton box: Chamyto® (apple nectar 200ml, grape nectar 200ml), Da fruta® (cashew 1000ml, guava 200ml and 1000ml, orange, mango and grape 1000ml), Del Valle® (orange nectar 1000ml and 1500ml, mango 1500ml, passion fruit 1000ml and grape 1000ml, Kapo orange 200ml, Kapo passion fruit 200m, Kapo strawberry 200ml), Disfrut® (cashew 200ml), Maguary® (orange and peach 1000ml), Sufresh® (cashew, guava, orange, mango, passion fruit, apple, strawberry, peach, grape 1000ml) Tial® (cashew, orange, grape 1000ml), Vigor® (orange 200ml)
**Juice concentrates and syrups**
Juice concentrate or syrup in plastic bottle: Açaicamp® (açaí, guarana with açaí 1000ml), Bela Ischia® (passion fruit 1000ml), Da Fruta® (cashew 500ml), Guaracamp® (diet guarana, guarana, guarana with blackcurrant 1000ml), Imbiara® (mango 1000ml and passion fruit 500ml), Jandaia® (cashew and passion fruit 500ml), Manguary® (cashew 500ml), Strong® (guarana 1000ml, Tropical® (blackcurrant 1000ml)
**Juice powder**
Juice powder in aluminum sachet: Fresh® (cashew, guava, orange, mango, passion fruit, grape 15g), Frisco® (pineapple, strawberry, grape 30g), Fresh® (cashew 30g), Mid® (pineapple 25g), Piraquê (passion fruit and peach 30g), Tang® (pineapple, lemon, passion fruit, strawberry, tangerine, grape 30g), Trink® (pineapple, pineapple with mint, guarana, lemon, peach 30g), Vilma® (grape 240g)
**Food supplements and complements**
Food supplements in can: Abbott® (Pediasure vanilla 400g), Danone® (Sustain Junior vanilla 350g), Olvebra® (Sustare Criança chocolate 480g), Sustagen® (Sustagen Kids chocolate and strawberry 380g)
**Seasonings and tomato products**
Seasoning tablet in carton box: Knorr® (bacon, vegetables, bacon with bay leaves 57g, beef and chicken 114g, Meu arroz (for rice), Meu arroz alho e cebola (garlic and onion for rice) and Meu feijão (for beans) 40g)
Ready seasoning in plastic bag: Ajinomoto® (beef, vegetables, for beans 50g), Maggi® (beef 50g)
Ready seasoning in plastic cup: Ajinomoto® (complete, without pepper 300g), Arisco® (garlic and salt 300g)

Photographs of the labels of each food group were analyzed to identify MCS. The MCS were defined according to the literature about communication through food labels and consumer perceptions, and organized according to the categorization in Chart 2^17–20^:

**Chart 2:** Description of marketing communication strategies on labels of food groups consumed by children under 5 years of age from the Brazilian National Health System (SUS). Rio de Janeiro, Brazil, 2014.

MCS on UPF labels	Description
Presence of characters and/or celebrities	Use of images of animals, figures or humanized foods, celebrities or cartoon characters in order to create a connection between the product and the person through confidence in their testimonies and search for status.
Emotional appeal	Connection between product consumption and a feeling of affection and protection.
Freebies offering	Indication of freebies offering, inside the packaging or attached to the label or describing the method of access.
Health appeal	Highlighting the nutritional composition of the product, the presence of certain ingredients, and messages that connect the product with health and well-being.
Sensory stimulation	Visual influence using colors, and reference to flavor, texture, and other sensory characteristics of the product.
Brand or slogan use	Brand/slogan is highlighted, used alone or in combination.
Promotional price	Reference to a lower price when compared to other products, or a higher quantity of the product for the same price.
Advertisement under advertisement	Advertisement of other products from the same brand, association of brands and indication of virtual spaces for more information.
Sustainability appeal	Use of messages about packaging recycling and brand actions related to environmental preservation.

MCS: marketing communication strategies; UPF: ultra-processed foods.

Label information was extracted in three stages: (1) first label analysis conducted by two researchers (each reviewed part of the labels); (2) review of the first analysis of all labels by a third researcher; and (3) resolution of doubts and inconsistencies between the two analyses by a new pair of researchers. The occurrences of each MCS were added to a Microsoft Excel 2010 spreadsheet and grouped according to the types of communication resources, which are used to support the strategies. They are based on thematic elements (e.g. cookery, health, nutrition, etc.) or visual elements (heart drawings, colors, packaging shape, etc.). Then, the types of resources were totaled by MCS and by product, and the occurrences of each MCS by food groups were added. The results were organized in two tables and a chart that show the percentage frequency of labels according to number of MCS per label, total and average frequency of MCS according to food group, frequency of MCS types on labels by food groups, systematization of MCS and types of communication resources with respective examples.

This study was approved by the Human Research Ethics Committee of the Municipal Health Department of the City of Rio de Janeiro (process n° 93/2013).

## RESULTS

In total, 2,792 marketing communication strategies were identified, ranging from 1 to 19 per label. The following distribution by category of MCS occurrence was observed: 33.6% of the labels showed 1 to 5 strategies; 57.9% showed 6 to 11 strategies; and 8.5% had 12 to 19 strategies, with an overall average of 7.2 strategies per label, ranging from 3.7 to 11.8. Soy drinks, flours, and breakfast cereals had the highest MCS averages per label (11.8, 11.2, and 10.8, respectively), and guarana soft drinks, margarines, and chocolate powders and similar products showed the lowest averages (4.8, 4.5, and 3.7) (Table 1).

**Table 1:** Total number of labels, percentage frequency of labels according to the number of marketing communication strategies (MCS) per label, total and average MCS according to food groups consumed by children under 5 years of age from the Brazilian National Health System (SUS). Rio de Janeiro, Brazil, 2014.

Food groups	Number of labels	Percentage frequency of labels according to number of MCS per label			Number of MCS	Average MCS per label
		1 to 5	6 to 11	12 to 19		
Soy drinks	13	15.4	23.1	61.5	154	11.8
Flours^a^	18	0	50	50	202	11.2
Breakfast cereals	4	0	50	50	43	10.8
Food supplements and complements^b^	5	0	100	0	48	9.6
Juice concentrates and syrups	14	7.1	85.7	7.1	116	8.3
Juice powder	24	20.8	79.2	0	193	8
Industrialized baby foods	2	0	100	0	16	8
Breads	7	0	100	0	55	7.9
Microwave popcorn	3	33.3	66.7	0	23	7.7
Seasonings	14	28.6	57.1	14.3	107	7.6
Soft drinks	10	0	100	0	76	7.6
Juice cartons	32	37.5	59.4	3.1	237	7.4
Ready-made cakes	8	12.5	87.5	0	58	7.3
Crackers and snacks	46	32.6	58.7	8.7	324	7
Dairy products	48	31.3	62.5	6.3	337	7
Peanuts	1	0	100	0	7	7
Cookies	50	42	56	2	312	6.2
Reconstituted meat products	10	60	30	10	61	6.1
Instant noodles	6	33.3	66.7	0	33	5.5
Sweets, candies, and goodies	57	54.4	43.9	1.8	311	5.5
Ready-made toasted manioc flour	3	66.7	33.3	0	15	5
Guarana drinks	5	80	20	0	24	4.8
Margarines	4	100	0	0	18	4.5
Chocolate powder and similar products	6	83.3	16.7	0	22	3.7
Total	390	33.6	57.9	8.5	2,792	7.2

MCS: marketing communication strategies.

a Mixtures of cereal flours.

b Formulations of nutrients, bioactive substances, enzymes or probiotics.

The strategies “sensory stimulation,” “health appeal,” “brand or slogan use,” and “advertisement under advertisement,” were observed in all food groups. “Freebies offering” and “promotional price” were identified in eight and six food groups, respectively. In breads; dairy products; and sweets, candies, and goodies, all nine types of MCS included in the study were identified. The groups with fewer types of MCS (n=5) were peanuts, instant noodles, and margarine (Table 2).

**Table 2:** Percentage frequency* of the type of marketing communication strategy adopted on labels according to the food groups consumed by children under 5 years of age from the Brazilian National Health System (SUS). Rio de Janeiro, Brazil, 2014.

Food groups	Senses^a^	Health^b^	Slogan^c^	Emotion^d^	Sustainability^e^	Advertising^f^	Characters^g^	Freebies^h^	Price^i^
(number of labels) (total strategies)	%	%	%	%	%	%	%	%	%
Soy drinks (13) (154)	10.4	45.5	11	10.4	16.2	5.8	0.6	0	0
Flours** (18) (202)	22.8	28.2	9.9	12.9	7.9	8.9	6.9	2.5	0
Breakfast cereals (4) (43)	27.9	16.3	14	11.6	11.6	7	7	4.7	0
Food supplements and complements*** (5) (48)	20.8	25	14.6	6.3	4.2	25	2.1	0	2.1
Juice concentrates and syrups (14) (116)	42.2	6.9	12.1	12.1	16.4	9.5	0.9	0	0
Juice powders (24) (193)	28.5	19.7	15	13	12.4	9.8	1	0.5	0
Industrialized baby foods (2) (16)	25	25	12.5	0	12.5	12.5	12.5	0	0
Breads (7) (55)	29.1	23.6	3.6	10.9	7.3	9.1	9.1	3.6	3.6
Microwave popcorn (3) (23)	30.4	17.4	17.4	17.4	4.3	13	0	0	0
Seasonings (14) (107)	28	9.3	15.9	23.4	15	7.5	0.9	0	0
Soft drinks (10) (76)	26.3	19.7	21.1	0	18.4	10.5	1.3	1.3	1.3
Juice cartons (32) (237)	19	24.5	13.5	13.5	13.5	13.1	3	0	0
Ready-made cakes (8) (58)	32.8	10.3	8.6	27.6	1.7	10.3	8.6	0	0
Crackers and snacks (46) (324)	27.2	14.5	17.9	14.5	14.2	8	3.1	0	0.6
Dairy products (48) (337)	30.6	20.8	11	7.4	9.8	11.9	7.4	0.9	0.3
Peanuts (1) (7)	14.3	14.3	28.6	28.6	0	14.3	0	0	0
Cookies (50) (312)	36.2	10.9	9.6	10.9	13.5	13.8	4.2	1	0
Reconstituted meat products (10) (61)	34.4	11.5	13.1	16.4	13.1	6.6	4.9	0	0
Instant noodles (6) (33)	45.5	9.1	12.1	21.2	0	12.1	0	0	0
Sweets, candies, and goodies (57) (311)	39.2	10.6	11.9	9.6	9.6	8.4	7.7	1.6	1.3
Ready-made toasted manioc flour (3) (15)	53.3	13.3	6.7	6.7	6.7	13.3	0	0	0
Guarana drinks (5) (24)	45.8	8.3	25	4.2	12.5	4.2	0	0	0
Margarines (4) (18)	27.8	22.2	16.7	16.7	0	16.7	0	0	0
Chocolate powders and similar products (6) (22)	18.2	18.2	9.1	22.7	13.6	4.5	13.6	0	0
**Total (390) (2,792)**	**29.4**	**18.2**	**12.9**	**12.1**	**11.7**	**10.2**	**4.3**	**0.8**	**0.4**

* Percentage calculated using the total marketing communication strategies found in each food group.

** Mixtures of cereal flours.

*** Formulations of nutrients, bioactive substances, enzymes or probiotics.

a Sensory stimulation.

b Health appeal.

c Brand or slogan use.

d Emotional appeal.

e Sustainability appeal.

f Advertisement under advertisement.

g Presence of characters and/or celebrities.

h Freebies offering.

i Promotional price.

Of total MCS identified on the labels, the most frequent were “sensory stimulation” (29.4%) and “health appeal” (18.2%); and the least frequent were “freebies offering” (0.8%) and “promotional price” (0.4%).

“Stimulation to the senses” was the most frequent strategy in 17 of total 24 food groups, ranging from 53.3% in the group of ready-made toasted manioc flour to 27.2% for crackers. “Health appeal” was the most frequent strategy in the following groups: soy drinks (45.5%), flours (28.2%), food supplements and complements (25%), and juice cartons (24.5%). In the group of food supplements and complements, “advertisement under advertisement” had the same frequency as “health appeal” (25%). “Emotional appeal” was the most frequent MCS in the group of chocolate powders and similar products (22.7%). In the group of industrialized baby foods, “health appeal” and “sensory stimulation” presented the same frequency (25%). In the group of peanuts, “emotional appeal” and “slogan use” also had the same frequency (28.6%) (Table 2).

Chart 3 shows all types of communication resources observed and their respective examples by MCS. The highest number of communication resources (n=13) was observed in “emotional appeal,” with “tradition, originality, exclusivity, and trust in the brand” as the most frequent type of resource.

**Chart 3:** Marketing communication strategy (MCS), type and example of communication resource present on labels of food groups consumed by children under 5 years of age from the Brazilian National Health System (SUS). Rio de Janeiro, Brazil, 2014.

Strategy	Type of communication resource	Example of communication resource
(number of types of resources)	(number of food groups)	(food groups)
Emotional appeal (13)	Tradition, originality, exclusivity and trust in the brand	Tradition since (year); “Traditional”; “Tradition and quality”; Affection with the family since (year); Original since (year); “1^st^ cream cheese in the world”; “The true…”; “The classic is back”; “Limited edition”; “Since when you were a child”; “Suitable for you”; “90 years in Brazil”; Thank-you phrase for choosing the product; “Original”; “The golden Japanese peanut!”; “#1 in sales in Brazil”; “Expert since 1984”; “Top 1 in Brazil.”
	16	(a) (b) (e) (g) (h) (i) (k) (l) (m) (o) (p) (q) (r) (t) (v) (z)
	Food preparation, ingredients, meal and flavor related or not to feelings	“It's ready, just heat it”; “Passionate about flavor”; “Home flavor is forever”; “A smile with every bite”; Dessert provides a happy and delicious moment after meals; “This pleasure has a new flavor”; “More flavor in your everyday life”; “Homemade taste”; “With selected ingredients”; “It's a lot of flavor and a lot more energy every day”; “The richest broth”; “Brings more peaches for you”; “The flavor that's worth it”; “Homemade cookies.”
	11	(a) (f) (g) (h) (i) (j) (n) (p) (r) (s) (v)
	Offers convenience	Highlihgting the ease of preparation; “Easy to prepare”; “Ready to drink.”
	10	(b) (d) (e) (f) (g) (i) (k) (m) (r) (s)
	Proximity to consumer	“Great option to complete your family's breakfast”; “One way or another, everyone eats it”; “Delicious pieces of life”; “Soy force to do more”; “How about looking at your kitchen in a different way? Find some adventure!”; “It's natural for you to like it”; “Natural and in your life”; “Savor every achievement”; “For us.”
	9	(e) (g) (h) (m) (p) (q) (r) (s) (v)
	Representation of people and bonds between them	Appeal to family and friends; Bond between mother and child during breastfeeding; Presence of children of different heights; “Enjoy this flavor with family and friends”; Presence of smiling mother and daughter on the front-of-packaging label; Happy child on the front-of-packaging label; Happy family on the back-of-packaging label; Image of son kissing his mother.
	7	(a) (b) (d) (h) (j) (l) (s)
	Barcode/nutrition table in a different format	Heart-shaped barcode; Bite-shaped barcode; Bar code with leaves; Pan-shaped barcode; Paw-shaped barcode; Film slate-shaped barcode.
	6	(a) (k) (m) (p) (v) (x)
	Use of an affectionate symbol on the label	Presence of a heart drawn around the brand; Image of hugging arms in the logo.
	6	(b) (e) (g) (h) (i) (j)
	Show of affection	Made with affection and tradition by the brand; “A child that sees a pediatrician feels better”; “It represents all the care and affection with which these products are prepared”; “A symbol of affection and love for you and your family”; “Made with love.”
	4	(a) (b) (g) (l)
	Social responsibility	“Child-friendly company”; “Social commitment”; “Abrinq Foundation - Child-friendly company.”
	6	(b) (h) (i) (o) (s) (v)
	Connection with fun	“Facing this challenge is pure fun”; “Indispensable energy and fun for your child's growth and development”; “Fun comes from inside”; “Energy to enjoy with friends.”
	4	(e) (h) (k) (n)
	Connection with social events	“Happy Holidays”; “The candy of the party.”
	2	(h) (i)
Emotional appeal (13)	Connection with meals throughout the day	“Start your day well”; “Made especially for your breakfast”; “Good morning”; “Energy for your morning”; “A delight that nourishes”; “Summer flavor.”
	4	(e) (h) (i) (r) (k)
	New format	
	1	
Sensory stimulation (6)	Product	Presence of an image of the product and/or ingredient on the packaging; Image with product presentation forms; Presence of a recipe that includes the product; Suggestions for consumption and/or preparation.
	24	(a) (b) (c) (d) (e) (f) (g) (h) (i) (j) (k) (l) (m) (n) (o) (p) (q) (r) (s) (t) (u) (v) (x) (z)
	Colors	Presence of primary colors on the packaging; Use of transparency; “It's perfect for adding color to foods and highlighting the flavor of your dishes.”
	22	(a) (b) (c) (d) (e) (f) (g) (h) (i) (j) (k) (l) (n) (p) (q) (r) (s) (t) (u) (v) (x) (z)
	Intensity	“More consistent”; “With selected ingredients”; “Tasty, delicious”; “Creamier”; “Tastier”; “Crunchier.”
	6	(e) (f) (h) (i) (k) (v)
	Texture	“New creamier formula”; “Creamy filling, crunchy cookie with chocolate drops”; “Easy to chew”; “Easy to chew, crunchy with filling”; “Soft and puffy”; “The perfect seasoning for sensational white loose rice”; “Melts in your mouth!”
	6	(e) (h) (i) (k) (p) (v)
	Flavor	“Lollipop with gum filling”; “Extra garlic!”; “Grape flavor and the sweetness of apple”; “It's already sweetened”; “More intense flavor.”
	5	(i) (p) (r) (s) (v)
	Onomatopoeia	“Nham strawberry?”; “Hummm.… SO DELICIOUS! This flavor will catch you.”
	2	(e) (v)
Health appeal (6)	Highlighting the nutritional composition of the product	Presence of vitamins and/or minerals and/or probiotics and/or fiber and/or omega 3 and 6.
	23	(d) (e) (g) (h) (h) (j) (k) (l) (m) (n) (q) (r) (s) (t) (z)
		Reduction of calories and/or sodium and/or total and trans fats and/or cholesterol; Highlights the presentation of fruits, vitamins, and iron; “With live lactobacilli paracasei”; “Free of trans fat”; “No preservatives”; “No added sugar”; “Free of transgenics.”
		(a) (b) (e) (g) (h) (i) (m) (n) (r) (u) (v) (x) (z)
		Made with animal protein/fruit or fruit juice/milk/small pieces of meat.
		(a) (b) (e) (h) (i) (k) (l) (n) (t) (u) (v)
		Nutrition information on the front-of-package label; Number of kcal per slice; “No sugar, less calories”; “Enjoy the right amount, 1 portion = 5 cookies”; “Same calories as an apple.”
		(b) (c) (e) (f) (g) (h) (i) (k) (m) (p) (q) (r) (s) (v) (z)
	Health tips and guidance	“Combination of vitamins and minerals that provides part of your child's nutritional needs”; Encouraging physical activity; Speech about healthy eating habits; “What is soy force?”; “Start the day with flavor and energy!!! Choco ball helps you quickly replenish your energy so you can spend it throughout the day. Include all types of food in your daily diet: fruits, vegetables, dairy products, and cereals. Reach out and get yours!!!”; “Don't add salt”; “Fruits are natural sources of vitamins”; “Have a healthier life”; “Eat well, live well!”
	12	(b) (f) (g) (i) (j) (m) (n) (p) (q) (r) (s) (u)
Health appeal (6)	Content/ingredient	“Rice+Corn+Wheat, a light combination of 3 cereals”; “40% more cocoa, rich in antioxidants”; “It's good to know: Do foods of plant origin contain lactose?”; “Rich in vitamin C”; “Made with sunflower oil.”
	6	(a) (g) (i) (m) (r) (v)
	Nutrition	“+ Energy with nutrition”; “Complements your diet”; “Nutritionally complete food”; “Energy”; Presents a puzzle with vitamin, saying that it complements the benefits of milk.
	7	(a) (b) (d) (e) (h) (i) (l)
	Emphasis on product formulation	“New formula”; “New recipe”; “Exclusive formula.”
	5	(a) (b) (e) (l) (v)
	Social responsibility	Quality seal – “Pro peanut ABICAB seal.”
	1	(o)
Advertising under advertising (3)	Availability of brand website, social media pages, QR code, and virtual store	(a) (b) (c) (d) (e) (f) (g) (h) (i) (j) (k) (l) (m) (n)(o) (p) (q) (r) (s) (t) (u) (v) (x)
	23	
	Encouragement to try other flavors available and other products of the same brand	(b) (d) (e) (f) (g) (h) (i) (o) (q) (r) (s) (v) (z)
	13	
	Association of two brands on the same product	“Tetrapak – Protects what is good”; “A Coca-Cola brand”; Danone logo on the label; Pepsico logo; Arcor logo.
	5	(e) (h) (m) (r) (v)
Sustainability appeal (2)	Recyclable product	Recyclable packaging seal; “Recycled paper”; “The paper of this packaging was produced with wood from FSC certified forests and other controlled sources”; “Recycle – everyone's commitment”; “Practice selective collection.”
	20	(a) (b) (c) (d) (e) (g) (h) (i) (l) (m) (n) (p) (q) (r) (s) (t) (u) (v) (x) (z)
	Environmental preservation	Message of environmental preservation; “Packaging produced from responsible sources”; “Practice selective collection”; “Preserve nature, recycle packaging”; “Keep your city clean”; “Nature thanks you”; “Respect life and nature”; “When you buy this product, you help take care of the world's forests”; “Preserve the environment. Save energy. Recycle materials.”
	15	(a) (b) (c) (e) (g) (h) (i) (l) (m) (n) (q) (r) (s) (t) (v)
Brand/slogan use (2)	Emphasis on the brand	(a) (c) (d) (e) (f) (g) (h) (i) (j) (k) (l) (m) (n) (o) (p) (q) (r) (s) (t) (u) (v) (z)
	22	
	Brand or product name associated with slogan or text	“With Neston, everything is more delicious, it becomes surreal”; “Whoever drinks Grapette repeats it”; “Sustagen + mom = allies for more complete nutrition”; “Nestlé is good for you”; “Start the day with Actimel”; “Why are Adria tarts so delicious? It's simple!”; “It's tasty to have Arisco at home”; “Santa Helena quality – Irresistible is living well”; “Yoki's deliciously fun snack”; “With Yoki popcorn, everything that is good gets better! Cinema is more cinema, football is more football, parties are more parties.”
	14	(b) (c) (d) (e) (f) (h) (i) (l) (m) (n) (o) (u) (v) (x)
Participation of celebrities and characters (2)	Character	Brand's own character; A current character; Animals and drawings.
	18	(a) (b) (d) (e) (f) (g) (h) (i) (j) (l) (l) (m) (n) (p) (r) (s) (u) (v)
	Humanized characters	Presence of humanized drawings; Animated human being; Humanized food.
	6	(b) (c) (e) (h) (i) (j)
Freebies offering (2)	Children's entertainment	Presence of game; Encouragement to access the website and play games available; Encouragement to visit the website for fun; Presence of comics; Presence of challenges.
	5	(b) (e) (g) (i) (n)
	Freebies and campaign	Freebies; “Campaign – Find and win it.”
	5	(c) (g) (h) (i) (s)
Promotional price (1)	Higher saving	“More savings”; “100g free”; “50% more”; “20% of the product free”; Suggested price; “Family size”; “+ cookies + Savings.”
	6	(c) (d) (e) (g) (i) (v)

Legends of food groups: (a) Reconstituted meat products; (b) Flours; (c) Soft drinks; (d) Food supplements and complements; (e) Dairy products; (f) Instant noodles; (g) Breads; (h) Cookies; (i) Sweets, candies, and goodies; (j) Margarines; (k) Ready-made cakes; (l) Chocolate powders and similar products; (m) Soy drinks; (n) Breakfast cereals; (o) Peanuts; (p) Seasonings; (q) Juice concentrates and syrups; (r) Juice cartons; (s) Juice powders; (t) Guarana drinks; (u) Industrialized baby foods; (v) Crackers; (x) Microwave popcorn; and (z) Ready-made toasted manioc flour.

The resource “product” (which includes the presence of product image, among others), related to the strategy of “sensory stimulation,” was used on the labels of all 24 food groups. The resources “highlighting aspects of the nutritional composition of the product” related to the strategy “health appeal” and “availability of brand website, social media pages, QR code, and virtual store” related to the strategy “advertisement under advertisement” were used on the labels of 23 of total 24 food groups. The resources “brand highlight” related to the strategy “brand/slogan use” (in 22 food groups), “colors” related to the strategy “sensory stimulation” (in 22 groups), and “recyclable product” related to the strategy “sustainability appeal” (in 20 groups) were also frequently used.

## DISCUSSION

The UPFs analyzed had an average of 7.2 MCS per label, ranging from one to 19 strategies; the most frequent ones were “sensory stimulation” and “health appeal.” In a study that analyzed 93 product labels and evaluated the quality of industrialized foods sold in supermarkets in the south area of Rio de Janeiro, up to eight MCS were identified in products for children^21^. The presence of these strategies on these labels can lead to brand or product loyalty^22^ and encourages children to persuade their parents to purchase these products^23^.

Exception for the group of guarana drinks, all groups of drinks had an average number of MCS equal to or greater than 7.4. Other studies using similar methods to that in our study, conducted with foods for children, obtained an average of 2.4 MCS per label for carbonated drinks or artificial juices in 2012 and an average of 4.4 MCS per label for juices, fruit nectars, and fruit drinks in 2013^21,24^.

A higher frequency of the strategy “sensory stimulation” agrees with the results of another study in which a third of the packages contained images or illustrations of *in natura* foods or the main ingredient^21^. In products for children sold in Uruguay, this was most common strategy in the group of cookies and sweets^20^.

In our analysis, the use of colors was very frequent. Considering that colors have a strong visual appeal, with subliminal messages and sensations, marketing and advertising professionals use them in packaging to highlight the product and attract the target audience. Children are generally attracted to colors like yellow, red, blue, and green. These colors influence stimulation, attention, and motivation to purchase and blue and green offer a feeling of calm and rest^25^. In Uruguay, the use of bright colors on food packaging for children is a strategy frequently used on candy and chocolate labels^20^. In a study evaluating the visual memory of cookie packaging and bags of snacks, the colors most remembered by children in their drawings were red, blue, yellow, and brown^26^.

Another aspect that must be considered is the presence of nutrition claims on UPF packaging, categorized as one of the types of communication resources related to the strategy “health appeal.” It was the second most frequent MCS, in agreement with the results of a study which showed that themes related to health, such as enriched foods, relationship between consumption and good health and growth, and nutrient completeness, had a high frequency of persuasive strategies on labels. The use of claims about added vitamins and minerals was recurrent in food products for children (dairy products, cookies, pastries, breakfast cereals, instant food, soft drinks, and juices)^20^.

Highlighting one or more nutrition claims on the food label can convey the misleading idea that it is a healthy food, producing a positive perception among consumers^27^. A study conducted in Brazil showed that nutrition claims on UPF packaging for children influenced the perception of children aged 8 to 10 years about the quality of the product as a whole^28^.

The adoption of sustainable diets has been encouraged by experts and institutions around the world, such as the Food and Agriculture Organization (FAO) of the United Nations^29^. In this sense, the “sustainability appeal” is among the MCS practiced by the food industries, as seen in our study. However, in most of these messages, the focus was not on the UPF production process, but on warning consumers regarding packaging disposal and environmental sustainability, for example.

Emphasis on the brand and slogan was the third most frequent MCS on labels. Brand is an important element of an advertisement, as it favorably influences the consumer's purchase decision and identifies the product, distinguishing it from the competition^26^. When a brand is promoted, all products of that company are favored^30^. In another study assessing the same topic, conducted with parents of children aged 2 to 12 years, brand and manufacturer recognition was the second main factor in the purchase decision of parents^31^.

Understanding how different marketing communication strategies reach adults and/or children still requires further analysis^32^. A scoping review identified studies that tracked types of communication strategies or measured their effectiveness and concluded that further studies are required assessing the persuasive power of these strategies depending on their type and the age of the children^14^. An experimental study with pairs of children and guardians revealed that advertisements of unhealthy products promoted favorable product perceptions and increased the preferences of guardians for the advertised products. It also noted that counter-advertising interventions can reinforce the resistance of parents and guardians to persuasive advertisement of these products and help them better evaluate unhealthy products^33^. Therefore, regulations for front-of-package labeling could constitute another formative element for families and become an education and health tool^34^. The freebies strategy was one of the least frequent MCS in this study, unlike findings of the literature, which indicate a frequency of 3.6% to 10% of the presence of freebies, games, and prize draws^21,24^. Our results may have underestimated the offer of freebies because the reference to freebies can be made via TV or the internet, or it can be a seasonal campaign and the study period did not coincide with it.

An alternative hypothesis would be an assessment of a tendency to reduction in the offer of freebies linked with products consumed by children. A potential explanation would be the implementation of Resolution no. 163 of March 13, 2014, issued by the Brazilian Council for the Rights of Children and Adolescents (Conanda), which provides regulations to prevent abusive advertising and marketing communications for children and adolescents, describing in Article 2 that abusive advertising refers to the promotions with distribution of prizes and collectible gifts or children appeal^35^. Abusive and misleading advertising to children violates the Federal Constitution, the Child and Adolescent Statute, and the Consumer Protection Code, since these legal provisions aim to protect children, who are considered vulnerable, due to their inability to identify commercial interests and persuasive aspects of advertisements^36^.

The development of regulations of front-of-package nutrition labeling is one of the priority measures of the regulatory agenda for the prevention and control of chronic non-communicable diseases^37^. In addition to providing the population with clear and more objective information about the real nutritional composition of UPFs, legal provisions can also ensure mechanisms to limit the presence of MCS on labels and the availability of these foods in food environments, as observed in Chile^38^. In Brazil, the technical standard approved by the National Health Surveillance Agency (Anvisa) to adapt front-of-package labeling of processed and ultra-processed foods (Collegiate Board Resolution – RDC 429/2020) was restricted to information about the nutritional composition of products and did not address the use of MCS on labels^39^.

The main study limitations were the fact that it was not possible to analyze 15% of all 459 products mentioned by the study participants as well as the partial analysis of 20.3% of all 390 labels included in the study. The analysis of a smaller number of products and partial sides of some labels may have led to errors (plus or minus) in the estimates of percentage and average frequencies of MCS analyzed in this study. Also important is the fact that 24-hour dietary recalls were applied between Tuesday and Friday, therefore not covering food consumption on weekends. It may have underestimated the number of UPFs consumed by the study children, assuming that the presence of UPFs is higher on weekends.

On the other hand, the analysis of labels of UPFs actually consumed by a probability sample of children from the SUS constitutes a strength of this study. When compared to the most frequent approach in the literature (analysis of products available in stores)^14^, this choice has the advantage of knowing the products actually consumed by children, regardless of whether the products were focused on them. Therefore, it can support the development of public policies for primary health care advice and food label regulation.

## CONCLUSION

In this study, MCS was identified on all labels of UPFs and industrialized baby foods consumed by children. It is important to adapt and improve the labels of foods for children and the general public, based on regulatory measures that prevent misleading and abusive practices. This is because the different types of MCS used on labels, especially those that stimulate the senses, emphasize aspects related to health, and highlight the brand can generate an exaggerated consumption of foods with low nutritional content, favoring an increase in childhood obesity and negative effects on health. Recognition of risks to child health and nutrition related to MCS reinforces that strict regulatory measures are required to protect consumers from massive exposure to MCS on food labels.

Also, public policies must encourage the dissemination of reliable information, based on dietary guidelines, to the entire population and through different media channels. The use of dietary guidelines by health, education, social care, and other professionals can support the development of food and nutritional education actions that address label reading and understanding, increase the critical sense of families, and promote more adequate and healthy eating habits.
