# Supplementary material for: Marketing communication strategies on labels of food products consumed by children
Source: Rev Saude Publica. 2023 Nov 17;57:92. doi: 10.11606/s1518-8787.2023057004614 (PMC10681567; doi:10.11606/s1518-8787.2023057004614)
Supplement: Translation

# Translation: Estratégias de comunicação mercadológica em rótulos de alimentos consumidos por crianças

**Authors:** Luciana Azevedo Maldonado, Silvia Cristina Farias, Kelly Veloso da Cruz, Bruna Pereira dos Santos, Luciana Maria Cerqueira Castro, Inês Rugani Ribeiro de Castro

**Affiliations:** 1 Rio de Janeiro RJ Brasil Universidade do Estado do Rio de Janeiro. Instituto de Nutrição. Departamento de Nutrição Social. Rio de Janeiro, RJ, Brasil.; 2 Rio de Janeiro RJ Brasil Universidade do Estado do Rio de Janeiro. Programa de Pós-graduação em Alimentação, Nutrição e Saúde. Rio de Janeiro, RJ, Brasil.; 3 Rio de Janeiro RJ Brasil Universidade do Estado do Rio de Janeiro. Instituto de Nutrição. Rio de Janeiro, RJ, Brasil.

**Keywords:** Saúde da Criança, Rotulagem de Alimentos, Publicidade de Alimentos, Nutrição da Criança

## Abstract

**OBJETIVO:**

Analisar estratégias de comunicação mercadológica (ECM) presentes em rótulos de produtos efetivamente consumidos por crianças menores de 5 anos usuárias do Sistema Único de Saúde na cidade do Rio de Janeiro.

**MÉTODOS:**

Foram analisados 390 rótulos de alimentos ultraprocessados e papas infantis industrializadas. Os produtos foram organizados por similaridade em 24 grupos. Realizou-se a análise das fotografias dos rótulos de cada grupo para a identificação das ECM, que foram categorizadas em: “presença de personagens e/ou celebridades”, “apelo emocional”, “oferta de brindes”, “apelo à saúde”, “estímulos aos sentidos”, “uso da marca ou slogan”, “preço promocional”, “propaganda sob propaganda” e “apelo à sustentabilidade”. Foram computadas: frequência percentual de rótulos segundo número de ECM por rótulo; frequência total e média de ECM segundo grupo de alimentos; frequência do tipo de ECM segundo grupo de alimentos; e recursos comunicacionais por tipo de ECM.

**RESULTADOS:**

Constataram-se de uma a 19 estratégias por rótulo e média de 7,2 ECM por rótulo, totalizando 2.792 ocorrências. As ECM “estímulo aos sentidos”, “apelo à saúde”, “uso da marca ou slogan” e “propaganda sob propaganda” foram observadas em todos os grupos de alimentos. Já “oferta de brindes” e “preço promocional” apareceram em oito e seis grupos de alimentos, respectivamente. Nos grupos pães, lácteos e doces, balas e guloseimas, foram identificados os nove tipos de ECM incluídos no estudo. Os grupos que apresentaram menos tipos de ECM (n = 5) foram: amendoins, macarrões instantâneos e margarinas. Do total de ECM identificadas nos rótulos, as mais recorrentes foram “estímulo aos sentidos” (29,4%) e “apelo à saúde” (18,2%); e as menos frequentes foram “oferta de brindes” (0,8%) e “preço promocional” (0,4%). A ECM “apelo emocional” apresentou a maior diversidade de recursos comunicacionais.

**CONCLUSÃO:**

São necessárias medidas regulatórias rigorosas que protejam o consumidor da massiva exposição às ECM em rótulos de alimentos.

## INTRODUÇÃO

O excesso de peso infantil tem apresentado expressivo aumento nas últimas décadas, atingindo 38,2 milhões de crianças menores de 5 anos no mundo^1,2^. Um importante fator para este fenômeno é o aumento do consumo de alimentos ultraprocessados (AUP). No Brasil, em 2019, a prevalência de consumo de AUP entre crianças de 6 a 23 e de 24 a 59 meses de idade foi de 80,5% e 93%, respectivamente^3^.

Os AUP são formulações industriais hiperpalatáveis que comprometem os hábitos alimentares saudáveis. Apresentam, em sua grande maioria, composição nutricional desbalanceada com altas quantidades de gorduras, açúcares, sal e aditivos alimentares com função cosmética, como emulsificantes, espessantes, corantes, edulcorantes, entre outros, não utilizados em preparações caseiras^4^. Além de ser associado à pior qualidade da dieta e à obesidade, o consumo desses alimentos também está relacionado a outros desfechos desfavoráveis em saúde, entre eles: cárie dentária, risco aumentado de doença cardiovascular, resistência à insulina, diabetes tipo 2 e deficiência de micronutrientes^5,6^.

A exposição de crianças a esses produtos é consequência da expansão da jornada de trabalho dos pais, incluindo o tempo de deslocamento entre a casa e o trabalho, da sobrecarga das mulheres com tarefas domésticas, do enfraquecimento das habilidades culinárias, da falta de opções mais saudáveis em escolas e outros ambientes alimentares^7–9^ e, também, do uso massivo de estratégias de marketing abusivas e enganosas sobre esses produtos, inclusive dirigidas ao público infantil^10^.

Dentre estas estratégias, destaca-se o uso das embalagens, tendo em vista sua função estratégica na comunicação entre o fabricante e o consumidor^11^. As embalagens são utilizadas para atrair os consumidores e estabelecer, no momento da escolha dos alimentos, um canal de comunicação direta sobre as supostas vantagens dos produtos. As indústrias alimentícias têm investido no desenvolvimento de design de embalagens, constituindo um potente canal de comunicação entre fabricante e consumidor cada vez mais utilizado para propagar significados e imagens que despertam a inclinação para aceitação, recompra e utilização dos produtos^11^.

As estratégias de marketing focam suas ações no público infantil pela grande capacidade de persuasão das crianças sobre as suas famílias^7^. A exposição a propagandas e outras estratégias de comunicação mercadológica (ECM) voltadas à promoção de AUP, veiculadas nos mais diversos meios de comunicação, principalmente televisão e internet, são ainda mais nocivas para crianças e adolescentes, pela falta de amadurecimento cognitivo para discernimento e compreensão da publicidade e de objetivos ocultos^12^. Por esse motivo, mundialmente, as propagandas de alimentos direcionadas ao público infantil e adolescentes têm sido objeto de políticas públicas, como as de regulação da publicidade e da rotulagem de alimentos^1,13^.

Estudos nesta temática têm pesquisado alimentos ou grupos alimentares específicos disponíveis em supermercados ou definidos com base em algum critério teórico^14^. Este estudo busca contribuir para o estado da arte sobre a publicidade em rótulos de alimentos por meio de outra abordagem: a análise das principais ECM presentes em rótulos de AUP e papas infantis industrializadas efetivamente consumidos por crianças menores de 5 anos de idade usuárias do SUS na cidade do Rio de Janeiro.

## MÉTODOS

O presente estudo deriva de um inquérito realizado em 2014 em amostra probabilística de 536 crianças com idade entre 6 e 59 meses, usuárias de unidades básicas de saúde da cidade do Rio de Janeiro. Nutricionistas treinados aplicaram recordatórios alimentares de 24 horas (R24h) a pais ou responsáveis pelas crianças. Foram coletadas informações referentes aos tipos de alimento, quantidades, formas de preparo, hora e local do consumo e, no caso de alimentos processados e AUP, suas respectivas marcas e sabores. A descrição detalhada dessa pesquisa está disponível em Anastácio et al.^15^

Após a verificação de todos os alimentos consumidos, os AUP foram identificados com base na classificação NOVA. As papas infantis industrializadas também foram incluídas por expressarem atributos semelhantes aos de AUP^16^. Em caso de dúvida, autores da classificação NOVA foram consultados. Foram selecionados para o estudo 459 produtos.

Entre março e dezembro de 2015, os rótulos dos AUP e papas infantis industrializadas, de acordo com sabor e/ou marca referidos pelo responsável no R24h, foram fotografados em estabelecimentos que comercializavam alimentos em diferentes bairros da cidade. Todas as embalagens de um mesmo produto disponíveis nestes estabelecimentos foram fotografadas (ex.: refrigerante em lata e em garrafa PET). Nesse caso, foi incluído somente um rótulo por produto, sendo incluído no banco de imagens aquele com maior número de ECM.

Antes de ir aos estabelecimentos, pesquisadores de campo foram treinados para fotografar todas as faces dos rótulos, de forma a prover as informações necessárias a todos os objetivos da pesquisa. O treinamento contemplou capacitação com exposição dialogada e atividade prática em estabelecimentos comerciais. As fotos foram realizadas com telefone celular e armazenadas em arquivos do programa Google Drive.

Foram analisados 390 rótulos de AUP e papas infantis industrializadas. Não foi possível analisar os rótulos de 69 (15%) dos 459 produtos pelos seguintes motivos: foto somente da face do rótulo que continha a tabela nutricional ou ausência de foto do rótulo (produto não localizado nos estabelecimentos visitados nem na internet). O banco de imagens apresentava, em geral, três a quatro fotos de diferentes faces de cada produto. Entretanto, não estavam disponíveis fotos de todas as faces do rótulo de 79 produtos (20,3% dos 390 estudados).

Os produtos foram organizados por similaridade em 24 grupos de alimentos, descritos no Quadro 1.

**Quadro 1:** Descrição dos produtos analisados por grupos de alimentos consumidos por crianças menores de 5 anos usuárias do SUS. Rio de Janeiro, Brasil, 2014.

**Produtos analisados por grupos de alimentos**
**Achocolatados em pó e similares**
Achocolatado em pó em pote de plástico: Showcau® (400g), Toddy® (200g, 400g e 800g)
Achocolatado em pó em lata de alumínio: Nescau® (400g)
Achocolatado em pó em sachê de plástico: Mágico® (200g e 400g)
Achocolatado Ovomaltine Flocos crocantes em sachê de plástico: Ovomaltine® (300g)
Saborizante em pó sabor morango em lata de alumínio: Nesquik® (380g)
**Amendoins**
Amendoim em embalagem plástica laminada: Mendorato (500g)
**Bebidas de soja**
Bebida em embalagem tetrapak: Ades® (de abacaxi, de laranja, de maçã, de morango, de pêssego, de soja, de uva 1000ml e de pêssego 200ml), Nestlé® (Sollys de maçã 1000ml e de laranja 200ml) Shefa® (original e de uva 1000ml), Sufresh® (de maçã 1000ml)
**Biscoitos doces**
Biscoito em embalagem de papelão: BelVita® (mel e cacau 30g)
Biscoito maizena em embalagem plástica: Duchen® (200g), Mabel® (400g), Marilan® (400g), Piraquê® (maizena e maria 200g), Vitarella® (tradicional e ao leite 400g)
Biscoito recheado em embalagem plástica laminada: Adria® (tortinha de chocolate, de chocolate branco, de chocolate ao leite, de chocolate tipo suíço, de morango e chocolate e de chocolate meio amargo 150g e de morango 160g, Plugados de chocolate e de limão 150g), Bauducco (de chocolate 130g e Gulosos 140g), Mabel® (de chocolate 140g), Marilan® (de morango 160g), Nestlé® (Bono de chocolate 140g, Passatempo de chocolate 140g e de morango 20g e 140g), Parati® (de chocolate 115g, de chocolate 140g), Piraquê® (de abacaxi, de chocolate e de limão e de morango 200g), Richester® (Amori de chocolate 140g), Trakinas® (de chocolate e de morango 143g, de morango e chocolate 136g)
Biscoito recheadinho goiabinha em embalagem plástica laminada: Bauducco® (140g) e Piraquê® (100g)
Biscoito rosquinha em embalagem plástica laminada: Mabel® (de banana e canela, de coco, de leite e de nata 400g), Piraquê® (de leite 400g)
Biscoito sem recheio em embalagem plástica laminada: Nestlé® (Passatempo de leite 150g)
Biscoito sequilhos em embalagem plástica laminada: Vovó Delma® (de leite 300g)
Biscoito tipo *cookie* em embalagem plástica laminada: Bauducco® (de chocolate (110g)
Biscoito tipo leite maltado em embalagem plástica laminada: Piraquê® (de chocolate crocante e natural 200g)
Biscoito tipo *wafer* em embalagem plástica laminada: Bauducco® (de chocolate 130g), Mirabel® (de chocolate e de morango 40g), Piraquê® (de chocolate 160g)
**Biscoitos salgados**
Biscoito água e sal em embalagem plástica laminada: Adria® (200g), Richester® (200g), Vitarella® (400g)
Biscoito em embalagem plástica laminada: Adria® (cream cracker 200g), Bauducco® (cracker levíssimo 200g), Club social® (de bacon 156g, integral 288g, original 155g e de pizza 141g), Elma Chips® (Baconzitos 55g, Cheetos de parmesão 45g, de queijo 59g e de requeijão 61g, Fandangos de queijo e de presunto 175g, Ruffles original 24g e 100g), Fofura® (de cebola, de churrasco e de queijo 100g), Mabel® (cracker 200g), Marilan® (cracker 400g, Pit stop de queijo e original 162g), Nestlé® (Cream cracker integral 170g, de gergelim 160g, Nesfit 126g), Piraquê® (cracker 200g, cracker integral 240g, de queijo 100g, Presuntinho 100g, Salgadinho 100g), Richester® (cracker 200g, Cream cracker gergelim 200g), Torcida® (de churrasco 50g, de queijo 80g e de pizza 80g), Triunfo® (cracker 200g), Vitarella® (cracker e cracker crocks 400g, Saltvip 156g)
Batata chips em embalagem plástica laminada: Elma Chips® (Sensações 45g),
Biscoito de polvilho em embalagem plástica: BC® (100g), Vale D'ouro® (100g)
**Bolos prontos**
Bolo em sachê de plástico: Ana Maria® (de cenoura e chocolate 40g e 80g; de baunilha com gotas de chocolate e recheio de chocolate 80g)
Bolo em sachê de plástico: Bauducco® (de brigadeiro 40g; de laranja 220g)
Bolo de morango em sachê de plástico: Panco® (70g)
Mistura para bolo sabor cenoura em sachê de plástico: Santa Amália® (400g)
Mistura para bolo sabor chocolate em sachê de plástico: Dona Benta® (400g)
Continua

**Cereais matinais**
Cereais matinais em caixa de papelão: Alca Foods® (300g), Nestlé® (270g e 330g), Kelloggs® (300g)
**Doces, balas e guloseimas**
Balas em saco plástico: Arcor® (7 Belo 150g), Butter Toffees® (de leite 750g), Dori® (de iogurte 600g), Embaré® (de leite tradicional 150g e de leite condensado 840g), Fini® (de morango 86g), Freegells® (Azedinha de morango e de azedinha sabor morango 420g), Gamadinho® (de doce de amendoim 600g), Juquinha® (de tutti frutti 300g), Peccin® (de morango, de iogurte, de laranja 600g)
Bala em caixa de papelão: Freegells® (de tangerina 31,7g), Mentos® (de fruta 428,8g e de *ice mint* 114g), Trident® (de menta 32g)
Bombom em saco de plástico: Garoto® (serenata do amor 19g)
Chiclete em caixa de papelão: Adams® (tutti frutti 280g)
Chocolate em caixa de papelão: Lacta® (Bis de chocolate 126g), Garoto® (Batom de chocolate ao leite 480g), Nestlé® (Alpino de chocolate 450g), Marisbel (de chocolate 300g)
Chocolate em saco de alumínio: Kinder Ovo® (de chocolate 20g)
Chocolate em lata de alumínio: Nestlé® (Moça doceria de brigadeiro 385g)
Chocolate em saco de plástico: Arcor® (Tortuguita de chocolate com cereal 140g), Cacau show® (de chocolate branco 20g), Dr. Oetker® (granulado macio 130g), Garoto® (chocolate branco 150g), Lacta® (Diamante negro 150g), M&M® (de amendoim e de chocolate 52g), Nestlé® (Classic de chocolate ao leite e de chocolate meio amargo 150g)
Creme de avelã: Nucita® (chocolate e avelã)
Doce de leite em pote de plástico: Pingo de leite® (500g)
Gelatina em caixa de papelão: Dr. Oetker® (morango 30g), Lual® (de framboesa 30g), Royal® (de framboesa, de limão e de morango 35g), Sol® (de limão, de morango e de uva 35g)
Geleia de mocotó em copo de vidro: Arisco® (de morango, natural e de tutti frutti 220g), Ducopo® (de morango e natural 180g),
Glucose de milho em pote de plástico: Karo® (350g)
Leite condensado em lata de alumínio: Nestlé® (Leite moça 395g)
Pirulito em saco de plástico: Peccin® (de tutti frutti 672g), Simas® (Pop de cereja 750g)
Sorvete: Bob's® (de casquinha de baunilha com calda de doce de leite), Kibom® (Chicabon, Creme e Cremosíssimo napolitano), McDonald's® (de casquinha de baunilha e chocolate)
**Farinhas**
Farinhas para preparo de mingaus saborizadas em latas de alumínio: Nestlé® (Neston de vitamina instantânea de morango, pera, banana e cereal e de mamão, maçã, banana, cereal; e Mucilon de milho 400g)
Farinhas para preparo de mingaus saborizadas em sachê de plástico: Nestlé® (Neston de vitamina instantânea de banana, mamão, maçã e cereal e de flocos 3 cereais 210g e Mucilon de arroz e de arroz e aveia e de multi cereais 230g), Nutri Day® (de arroz 200g), Nutrilon® (de aveia 230g), Vitalon® (de 6 cereais 200g)
Farinha láctea em sachê de plástico: Nestlé® (210g)
Farinha para preparo de mingaus sabor creme de arroz com adição de vitaminas e minerais em saco plástico: Chinezinho® (200g), Yoki® (200g)
Farinha para preparo de mingaus à base de amido de milho em caixa de papelão: Cremogema Maizena® (tradicional, de morango, de banana, de chocolate 200g)
**Farofas prontas**
Farofa pronta em saco laminado: Sinhá® (de bacon temperada e de mandioca temperada 250g), Yoki® (de mandioca temperada 500g)
**Lácteos**
Bebida láctea saborizada em caixa de papelão: Elegê® (de morango 200ml), Del Valle® (de chocolate 200ml), Nestlé® (Mucilon prontinho de laranja maçã e pera e de banana e pêssego 190ml), Toddynho® (de chocolate 200ml)
Bebida láctea saborizada em garrafa de plástico: Bialini® (de morango 800g), Danone® (de morango 170g e de coco 900g), Elegê® (de morango 900g), Itambé® (de morango 600g), Nestlé® (de maçã e banana 170g), Vigor® (de morango 180g), Yofruta® (de morango 170g)
Cream cheese em pote de plástico: Philadelphia® (150g)
Iogurtes saborizados em bandejas de plástico: Batavo® (Grego de morango 400g), Danone® (Danoninho de morango e de banana, morango e maçã verde 360g, Danoninho de morango e banana 540g), Elegê® (queijo petit-suisse de morango (Bob Esponja) 320g), Itambé® (queijo petit-suisse de morango e de morango e banana 360g), Nestlé® (Chambinho de morango 320g, Ninho de morango e de três sabores 600g e de morango 540g), Paulista® (queijo petit-suisse de morango (galinha pintadinha) 330g), Vigor® (de chocolate 200g, grego kids morango 360g e de morango e de morango e vitamina de frutas 540g)
Iogurtes saborizados em potes de plástico: Frimesa® (de morango 165g), Paulista® (Grego com calda de morango 95g) e Vigor® (Grego de baunilha e calda de morango 95g, Grego tradicional e de baunilha 100g e de laranja, cenoura e mel 170g);
Leite fermentado em caixa de papelão: Elegê® (80ml)
Leite fermentado em garrafa de plástico: Actimel® (600g), Batavo® (480g), Danone® (450g e 600g), Nestlé® (450g), Paulista® (450g), Yakult® (480g)
Polenguinho® em caixa de papelão: (Com 8 unidades de 17g cada)
Requeijão cremoso em copo de vidro: Itambé® (220g)
Requeijão cremoso em pote de plástico: Godam® (light 200g), Polenghi® (normal e light 200g), Sadia® (200g), Vigor® (200g)
**Macarrões instantâneos**
Macarrão instantâneo em saco plástico: Maggi® (de carne e de galinha 85g), Nissin® (de galinha caipira e de galinha 85g), Richester® (de carne 85g), Vigor® (de carne 85g)
**Margarinas**
Margarina em pote de plástico: Delícia® (500g), Doriana® (250g e 500g) e Qualy® (500g)
**Pães**
Pão para hambúrguer em saco plástico: Plus Vita® (480g)
Pão tipo bisnaguinha em saco plástico: Panco® (300g), Plus Vita® (360g), Wickbold® (300g)
Pão de forma em saco plástico: Plus Vita® (500g)
Torrada em saco plástico: Bauducco® (160g), Marilan® (150g), Wickbold® (140g)
**Papas infantis industrializadas**
Papas industrializadas em copos de vidro: Nestlé® (de frutas sortidas 120g e de arroz, feijão e carne 170g)
**Pipocas de micro-ondas**
Pipoca de micro-ondas em sachê de plástico: Yoki® (sabor caramelo 160g, sabor manteiga 100g e sabor natural 100g)
**Produtos de carne reconstituída**
Hambúrguer em caixa de papelão: Perdigão® (de carne de frango e bovina 672g), Sadia® (de carne bovina 672g)
Linguiça calabresa em saco de plástico: Perdigão® (400g) e Seara® (400g)
Mortadela em saco de plástico: Perdigão® (1000g)
Nuggets em caixa de papelão: Sadia® (tradicional 300g), Seara® (tradicional Turma da Mônica 300g)
Presunto em saco de plástico: Sadia® (200g)
Salsicha em saco de plástico: Perdigão® (500g) e Sadia® (500g)
**Refrigerantes**
Refrigerante em garrafa de plástico: Coca-cola® (2000ml), Dolly® (de guaraná 350ml), Fanta® (de laranja e de uva 2000ml), Grapette® (de framboesa 290ml), Pepsi® (2500ml), Schin® (de guaraná 2000ml), Sprite® (de limão 2000ml), Tobi® (de cola e de guaraná 350ml)
**Refrescos de guaraná**
Refresco em copo de plástico: Açaicamp® (de açaí 285ml), Ativ plus® (de guaraná 290ml), Guaracamp® (de guaraná 285ml), Guaraplus® (de guaraná 290ml), Guaravita® (de guaraná 290ml)
**Sucos de caixa**
Suco em caixa de papelão: Chamyto® (de néctar de maçã 200ml, de néctar de uva 200ml), Da fruta® (de caju 1000ml, de goiaba 200ml e 1000ml, de laranja, de manga e de uva 1000ml), Del Valle® (de néctar de laranja 1000ml e 1500ml, de manga 1500ml, de maracujá 1000ml e de uva 1000ml, Kapo de laranja 200ml, Kapo de maracujá 200m, Kapo de morango 200mll), Disfrut® (de caju 200ml), Manguary® (de laranja e de pêssego 1000ml), Sufresh® (de caju, de goiaba, de laranja, de manga, de maracujá, de maçã, de morango, de pêssego e de uva 1000ml) Tial® (de caju, de laranja e de uva 1000ml), Vigor® (de laranja 200ml)
**Sucos concentrados e xaropes**
Suco concentrado ou xarope em garrafa de plástico: Açaicamp® (de açaí, de guaraná com açaí 1000ml), Bela Ischia® (de maracujá 1000ml), Da fruta® (de caju 500ml), Guaracamp® (diet de guaraná, de guaraná, de guaraná com groselha 1000ml)), Imbiara® (de manga 1000ml e de maracujá 500ml), Jandaia® (de caju e de maracujá 500ml), Maguary® (de caju 500ml), Strong® (de guaraná 1000ml, Tropical® (de groselha 1000ml)
**Sucos em pó**
Suco em pó em sachê de alumínio: Fresh® (de caju, de goiaba, de laranja, de manga, de maracujá e de uva 15g), Frisco® (de abacaxi, de morango e de uva 30g), Fresh® (de caju 30g), Mid® (de abacaxi 25g), Piraquê (de maracujá e de pêssego 30g), Tang® (de abacaxi, de limão, de maracujá, de morango, de tangerina e de uva 30g), Trink® (de abacaxi, abacaxi com hortelã, de guaraná, de limão e de pêssego 30g), Vilma® (de uva 240g)
**Suplementos e complementos alimentares**
Suplementos alimentares em lata: Abbott® (Pediasure de baunilha 400g), Danone® (Sustain junior de baunilha 350g), Olvebra® (Sustare criança de chocolate 480g), Sustagen® (Sustagen kids de chocolate e de morango 380g)
**Temperos e atomatados**
Tablete de tempero em caixa de papelão: Knorr® (de bacon, de legumes, de bacon com louro 57g, de carne e de galinha 114g, meu arroz, meu arroz alho e cebola e meu feijão 40g)
Tempero pronto em saco de plástico: Ajinomoto® (de carne, de feijão e de legumes 50g), Maggi® (de carnes 50g)
Tempero pronto em copo plástico: Ajinomoto® (completo sem pimenta 300g), Arisco® (de alho e sal 300g)

Realizou-se a análise das fotografias dos rótulos de cada grupo de alimentos para a identificação das ECM. Estas foram definidas com base em literatura referente à comunicação por meio de rótulos de alimentos e percepções dos consumidores, e organizadas conforme categorização apresentada no Quadro 2^17–20^:

**Quadro 2:** Descrição das estratégias de comunicação mercadológica presentes em rótulos de grupos de alimentos consumidos por crianças menores de 5 anos usuárias do SUS. Rio de Janeiro, Brasil, 2014.

ECM presente nos rótulos de AUP	Descrição
Presença de personagens e/ou celebridades	Uso de imagens de animais, figuras ou alimentos humanizados, de celebridades ou personagens de desenhos animados, a fim de criar um vínculo entre o produto e a pessoa, pela confiança em seu depoimento e pela busca de status
Apelo emocional	Articulação entre o consumo daquele produto a sensação de afeto e proteção
Oferta de brindes	Indicação no rótulo da oferta de brinde, seja no interior da embalagem, anexado ao rótulo ou por descrição da forma de acesso
Apelo à saúde	Valorização da composição nutricional do produto, da presença de determinados ingredientes, e mensagens que vinculam o produto a saúde e bem-estar
Estímulo aos sentidos	Influência pela visão, através das cores, e por alusão ao sabor, texturas e outras características sensoriais do produto
Uso da marca ou slogan	Utilização em destaque, de forma isolada ou combinada
Preço promocional	Referência a preço mais baixo, quando comparado a outros produtos, ou a maior quantidade do produto pelo mesmo preço
Propaganda sob propaganda	Divulgação de outros produtos da mesma marca, associações entre marcas e indicação de espaços virtuais para mais informações
Apelo à sustentabilidade	Uso de mensagens sobre reciclagem de embalagens e de ações da marca para a preservação do meio ambiente

ECM: estratégia de comunicação mercadológica. AUP: alimentos ultraprocessados.

A extração das informações dos rótulos foi realizada em três etapas: 1) primeira análise dos rótulos, realizada por duas pesquisadoras (cada uma revisou parte dos rótulos); 2) revisão da primeira análise de todos os rótulos por uma terceira pesquisadora; e 3) resolução das dúvidas e inconsistências entre as duas análises por uma nova dupla de pesquisadoras. As ocorrências de cada ECM foram registradas em planilha do software Microsoft Excel versão 2010 e agrupadas de acordo com os tipos de recursos comunicacionais, que são as formas pelas quais as ECM se concretizam. Eles são baseados em elementos temáticos (ex.: culinária, saúde, nutrição etc.) ou visuais (desenho de coração, cores, formato da embalagem etc.). Em seguida, procedeu-se à totalização dos tipos de recursos por ECM e por produto e ao somatório de ocorrências de cada ECM por grupos de alimentos. Os resultados foram organizados em duas tabelas e um quadro que abarcam: frequência percentual de rótulos segundo número de ECM por rótulo, frequência total e média de ECM segundo grupo de alimentos; frequência dos tipos de ECM nos rótulos por grupos de alimentos; sistematização das ECM e dos tipos de recursos comunicacionais, com respectivos exemplos.

A pesquisa foi aprovada pelo Comitê de Ética em Pesquisa com Seres Humanos da Secretaria Municipal de Saúde da Prefeitura da Cidade do Rio de Janeiro (Processo n° 93/2013).

## RESULTADOS

Foram identificadas 2.792 ECM, variando de uma a 19 por rótulo. Foi observada a seguinte distribuição por categoria de ocorrência de ECM: 33,6% dos rótulos continham entre um e cinco ECM; 57,9% apresentaram entre seis e 11 ECM; e 8,5% incluíam de 12 a 19 ECM, com média geral de 7,2 estratégias por rótulo, variando de 3,7 a 11,8. Bebidas de soja, farinhas e cereais matinais apresentaram as maiores médias de ECM por rótulo (11,8, 11,2 e 10,8, respectivamente) e refrescos de guaraná, margarinas e achocolatados em pó e similares, as menores médias (4,8, 4,5 e 3,7) (Tabela 1).

**Tabela 1:** Total de rótulos, frequência percentual de rótulos segundo número de estratégias de comunicação mercadológica (ECM) por rótulo, total e média de ECM segundo grupo de alimentos consumidos por crianças menores de 5 anos de idade usuárias do SUS. Rio de Janeiro, Brasil, 2014.

Grupo de alimentos	N° de rótulos	Frequência % de rótulos por número de ECM por rótulo			N° de ECM	Média de ECM por rótulo
		1 a 5	6 a 11	12 a 19		
Bebidas de soja	13	15,4	23,1	61,5	154	11,8
Farinhas^a^	18	0	50	50	202	11,2
Cereais matinais	4	0	50	50	43	10,8
Suplementos e complementos alimentares^b^	5	0	100	0	48	9,6
Sucos concentrados e xaropes	14	7,1	85,7	7,1	116	8,3
Sucos em pó	24	20,8	79,2	0	193	8
Papas infantis industrializadas	2	0	100	0	16	8
Pães	7	0	100	0	55	7,9
Pipocas de micro-ondas	3	33,3	66,7	0	23	7,7
Temperos	14	28,6	57,1	14,3	107	7,6
Refrigerantes	10	0	100	0	76	7,6
Sucos de caixa	32	37,5	59,4	3,1	237	7,4
Bolos prontos	8	12,5	87,5	0	58	7,3
Biscoitos salgados	46	32,6	58,7	8,7	324	7
Lácteos	48	31,3	62,5	6,3	337	7
Amendoins	1	0	100	0	7	7
Biscoitos doces	50	42	56	2	312	6,2
Produtos de carne reconstituída	10	60	30	10	61	6,1
Macarrões instantâneos	6	33,3	66,7	0	33	5,5
Doces, balas e guloseimas	57	54,4	43,9	1,8	311	5,5
Farofas prontas	3	66,7	33,3	0	15	5
Refrescos de guaraná	5	80	20	0	24	4,8
Margarinas	4	100	0	0	18	4,5
Achocolatados em pó e similares	6	83,3	16,7	0	22	3,7
Total dos grupos	390	33,6	57,9	8,5	2.792	7,2

ECM: estratégia de comunicação mercadológica.

a Misturas à base de farinhas de cereais.

b Formulações de nutrientes, substâncias bioativas, enzimas ou probióticos.

As ECM “estímulo aos sentidos”, “apelo à saúde”, “uso da marca ou slogan” e “propaganda sob propaganda” foram observadas em todos os grupos de alimentos. Já “oferta de brindes” e “preço promocional” ocorreram em oito e seis grupos de alimentos, respectivamente. Nos grupos pães, lácteos e doces, balas e guloseimas, foram identificados os nove tipos de ECM incluídos no estudo. Os grupos que apresentaram menos tipos de ECM (n = 5) foram: amendoins, macarrões instantâneos e margarinas (Tabela 2).

**Tabela 2:** Frequência percentual* do tipo de estratégia de comunicação mercadológica adotado nos rótulos segundo grupos de alimentos consumidos por crianças menores de 5 anos de idade usuárias do SUS. Rio de Janeiro, Brasil, 2014.

Grupo de Alimentos	Sentidos^a^	Saúde^b^	Slogan^c^	Emoção^d^	Sustentabilidade^e^	Propaganda^f^	Personagem^g^	Brinde^h^	Preço^i^
(n° de rótulos) (total de estratégias)	%	%	%	%	%	%	%	%	%
Bebidas de soja (13) (154)	10,4	45,5	11	10,4	16,2	5,8	0,6	0	0
Farinhas** (18) (202)	22,8	28,2	9,9	12,9	7,9	8,9	6,9	2,5	0
Cereais matinais (4) (43)	27,9	16,3	14	11,6	11,6	7	7	4,7	0
Suplementos e complementos alimentares*** (5) (48)	20,8	25	14,6	6,3	4,2	25	2,1	0	2,1
Sucos concentrados e xaropes (14) (116)	42,2	6,9	12,1	12,1	16,4	9,5	0,9	0	0
Sucos em pó (24) (193)	28,5	19,7	15	13	12,4	9,8	1	0,5	0
Papas infantis industrializadas (2) (16)	25	25	12,5	0	12,5	12,5	12,5	0	0
Pães (7) (55)	29,1	23,6	3,6	10,9	7,3	9,1	9,1	3,6	3,6
Pipocas de micro-ondas (3) (23)	30,4	17,4	17,4	17,4	4,3	13	0	0	0
Temperos (14) (107)	28	9,3	15,9	23,4	15	7,5	0,9	0	0
Refrigerantes (10) (76)	26,3	19,7	21,1	0	18,4	10,5	1,3	1,3	1,3
Sucos de caixa (32) (237)	19	24,5	13,5	13,5	13,5	13,1	3	0	0
Bolos prontos (8) (58)	32,8	10,3	8,6	27,6	1,7	10,3	8,6	0	0
Biscoitos salgados (46) (324)	27,2	14,5	17,9	14,5	14,2	8	3,1	0	0,6
Lácteos (48) (337)	30,6	20,8	11	7,4	9,8	11,9	7,4	0,9	0,3
Amendoins (1) (7)	14,3	14,3	28,6	28,6	0	14,3	0	0	0
Biscoitos doces (50) (312)	36,2	10,9	9,6	10,9	13,5	13,8	4,2	1	0
Produtos de carne reconstituída (10) (61)	34,4	11,5	13,1	16,4	13,1	6,6	4,9	0	0
Macarrões instantâneos (6) (33)	45,5	9,1	12,1	21,2	0	12,1	0	0	0
Doces, balas e guloseimas (57) (311)	39,2	10,6	11,9	9,6	9,6	8,4	7,7	1,6	1,3
Farofas prontas (3) (15)	53,3	13,3	6,7	6,7	6,7	13,3	0	0	0
Refrescos de guaraná (5) (24)	45,8	8,3	25	4,2	12,5	4,2	0	0	0
Margarinas (4) (18)	27,8	22,2	16,7	16,7	0	16,7	0	0	0
Achocolatados em pó e similares (6) (22)	18,2	18,2	9,1	22,7	13,6	4,5	13,6	0	0
**Total geral (390) (2.792)**	**29,4**	**18,2**	**12,9**	**12,1**	**11,7**	**10,2**	**4,3**	**0,8**	**0,4**

* Percentual calculado sobre o total de estratégias de comunicação mercadológica encontradas em cada um dos grupos de alimentos.

** Misturas à base de farinhas de cereais.

*** Formulações de nutrientes, substâncias bioativas, enzimas ou probióticos.

a Estímulo aos sentidos.

b Apelo à saúde.

c Uso da marca ou slogan.

d Apelo emocional.

e Apelo à sustentabilidade.

f Propaganda sob propaganda.

g Presença de personagens e/ou celebridades.

h Oferta de brindes.

i Preço promocional.

Do total de ECM identificadas nos rótulos, as mais recorrentes foram “estímulo aos sentidos” (29,4%) e “apelo à saúde” (18,2%); e as menos frequentes foram “oferta de brindes” (0,8%) e “preço promocional” (0,4%).

A ECM “estímulo aos sentidos” foi a mais frequente em 17 dos 24 grupos de alimentos, variando de 53,3% no grupo de farofas prontas a 27,2% entre biscoitos salgados. A ECM “apelo à saúde” foi a mais frequente nos seguintes grupos: bebidas de soja (45,5%), farinhas (28,2%), suplementos e complementos alimentares (25%) e sucos de caixa (24,5%). No grupo dos suplementos e complementos alimentares, a ECM “propaganda sob propaganda” apresentou a mesma frequência que “apelo à saúde” (25%). A ECM “apelo emocional” foi a mais frequente no grupo achocolatados em pó e similares (22,7%). No grupo de papas infantis industrializadas, as estratégias “apelo à saúde” e “estímulo aos sentidos” apresentaram a mesma frequência (25%). O mesmo aconteceu no grupo amendoins com as ECM “apelo emocional” e “uso de slogan” (28,6%) (Tabela 2).

O Quadro 3 sistematiza todos os tipos de recursos comunicacionais observados e seus respectivos exemplos por ECM. A maior diversidade de recursos comunicacionais (n = 13) foi observada na ECM “apelo emocional”, sendo “tradição, originalidade, exclusividade e confiança na marca” o tipo de recurso mais recorrente.

**Quadro 3:** Estratégia de Comunicação Mercadológica (ECM), tipo e exemplo de recurso comunicacional presentes em rótulos de grupos de alimentos consumidos por crianças menores de 5 anos usuárias do SUS. Rio de Janeiro, Brasil, 2014.

Estratégia	Tipo de recurso comunicacional	Exemplo de recurso comunicacional
(número de tipos de recursos)	(número de grupos de alimentos)	(grupos de alimentos)
Apelo emocional (13)	Tradição, originalidade, exclusividade e confiança na marca	Tradição desde (ano); “Tradicional”; “Tradição e qualidade”; Carinho com a família desde (ano); Original desde (ano); “1° *cream cheese* do mundo”; “O verdadeiro”; “O clássico voltou”; “Edição limitada”; “Desde sua infância”; “Na sua medida”; “90 anos no Brasil”; Frase de agradecimento pela escolha do produto; “Original”; “O amendoim japonês dourado!”; N° 1 em vendas no Brasil”; “Desde 1984 é especialista”; “A 1ᵃ do Brasil”
	16	(a) (b) (e) (g) (h) (i) (k) (l) (m) (o) (p) (q) (r) (t) (v) (z)
	Preparação culinária, ingredientes, refeição e sabor relacionado ou não com sentimentos	“Já vem pronto, é só aquecer”; “Apaixonados por sabor”; “Sabor de casa é para sempre”; “A cada mordida um sorriso”; Sobremesa proporciona um momento alegre e gostoso após as refeições; “Esse prazer tem novo sabor”; “Mais sabor no seu dia a dia”; “Gostinho caseiro”; “Com ingredientes selecionados”; “É muito sabor e muito mais energia todos os dias”; “O caldo mais encorpado”; “Traz mais pêssego para você”; “O sabor que vale a pena”; “Biscoito caseiro”
	11	(a) (f) (g) (h) (i) (j) (n) (p) (r) (s) (v)
	Praticidade oferecida	Apelo por praticidade de preparo; “Fácil de preparar”; “Pronto para beber”
	10	(b) (d) (e) (f) (g) (i) (k) (m) (r) (s)
	Aproximação com o consumidor	“Ótima opção para completar o café da manhã da sua família”; “De um jeito ou de outro todo mundo come”; “Pedaços gostosos da vida”; “Soy force força para fazer mais”; “Que tal olhar sua cozinha de um jeito diferente? Aventure-se!”; “É natural você gostar”; “Natural e na sua vida”; “Saboreie cada conquista”; “Pra nós”
	9	(e) (g) (h) (m) (p) (q) (r) (s) (v)
	Representação de pessoas e de vínculo entre si	Apelo a família e amigos; Vínculo entre mãe e filho na amamentação; Presença de crianças de diferentes alturas; “Curta esse sabor com a família e amigos”; Presença de mãe e filha sorridentes na parte frontal do rótulo; Criança feliz na parte frontal do rótulo; Imagem de família feliz na parte posterior do rótulo; Imagem de filho beijando a mãe
	7	(a) (b) (d) (h) (j) (l) (s)
	Código de barras/tabela nutricional em formato diferenciado	Código de barras em formato de coração; Código de barras em formato de mordida; Código de barra com folhinhas; Código de barras em formato de panela; Código de barra em formato de pata; Código de barras em formato de claquete de cinema
	6	(a) (k) (m) (p) (v) (x)
	Uso de símbolo afetivo no rótulo	Presença de coração em volta da marca; Imagem de braços em formato de abraço na logo
	6	(b) (e) (g) (h) (i) (j)
	Demonstração de afeto	Feito com carinho e tradição pela marca; “Quem vai ao pediatra volta tranquilo”; “Ele representa todos o cuidado e carinho com que seus produtos são preparados”; “Um símbolo de carinho e amor para você e sua família”; “Feito com amor”
	4	(a) (b) (g) (l)
	Responsabilidade social	“Empresa amiga da criança”; “Compromisso social”; “Fundação Abrinq-Empresa amiga da Criança”
	6	(b) (h) (i) (o) (s) (v)
	Promoção de diversão	“Encarar esse desafio é pura diversão”; “Energia e diversão indispensáveis para crescimento e desenvolvimento do seu filho”; “Diversão vem de dentro”; “Energia para curtir com a galera”
	4	(e) (h) (k) (n)
	Relação com eventos sociais	“Boas festas”; “A bala da festa”
	2	(h) (i)
Apelo emocional (13)	Relação com refeições ao longo do dia	“Comece bem o seu dia”; “Feito especialmente para seu café da manhã”; “Bom dia”; “Energia para sua manhã”;’ “A delícia que alimenta”; “Sabor de verão”
	4	(e) (h) (i) (r) (k)
	Novo formato	
	1	
Estímulo aos sentidos (6)	Produto	Presença de imagem do produto e ou ingrediente ilustrado na embalagem; Imagem com formas de apresentação do produto; Presença de Receita que utiliza o produto; Sugestão de consumo e ou preparo
	24	(a) (b) (c) (d) (e) (f) (g) (h) (i) (j) (k) (l) (m) (n) (o) (p) (q) (r) (s) (t) (u) (v) (x) (z)
	Cores	Presença de cores primárias na embalagem; Uso de transparência; “É perfeito para dar cor e destacar o sabor de seus pratos”
	22	(a) (b) (c) (d) (e) (f) (g) (h) (i) (j) (k) (l) (n) (p) (q) (r) (s) (t) (u) (v) (x) (z)
	Intensidade	“Mais consistente”; “Com ingredientes selecionados”; “O gostoso, o delicioso”; “+ cremoso”; “Mais saboroso”; “Mais crocante”
	6	(e) (f) (h) (i) (k) (v)
	Textura	“Nova fórmula mais cremosa”; “Recheio cremoso, biscoito crocante e gota de chocolate”; “Mastigável”; “Mastigável, crocante e recheado”; “Macio e fofinho”; “O tempero perfeito para um arroz sensacional, branquinho e soltinho”; “Derrete na boca!”
	6	(e) (h) (i) (k) (p) (v)
	Sabor	“Pirulito com recheio de chiclete”; “Extra alho!”; “Gostinho da uva e o docinho da maçã”; “Já vem adoçado”; “Sabor mais intenso”
	5	(i) (p) (r) (s) (v)
	Onomatopeia	“Nham morango?”; “hummm.… QUE DELÍCIA! O sabor que vai pegar você.”
	2	(e) (v)
Apelo à saúde (6)	Destaque a aspectos da composição nutricional do produto	Presença de vitaminas e/ou minerais e/ou probióticos e/ou fibras e/ou ômega 3 e 6
	23	(d) (e) (g) (h) (h) (j) (k) (l) (m) (n) (q) (r) (s) (t) (z)
		Redução de calorias e/ou sódio e/ou gorduras totais e trans e/ou colesterol; Destaque na apresentação de frutas, vitaminas e ferro; “Com lactobacilos vivos paracasei”; “Livre de gordura trans”; “Sem conservantes”; “Sem adição de açúcar”; “Livre de transgênicos”
		(a) (b) (e) (g) (h) (i) (m) (n) (r) (u) (v) (x) (z)
		Feito com proteína animal/frutas ou suco de frutas/leite/pequenos pedaços de carne
		(a) (b) (e) (h) (i) (k) (l) (n) (t) (u) (v)
		Informação nutricional na parte frontal do rótulo; Número de kcal por fatia; “Sem açúcar, menos caloria”; “Galera curtam na medida certa 1 porção = 5 biscoitos”; “Mesma caloria que uma maçã”
		(b) (c) (e) (f) (g) (h) (i) (k) (m) (p) (q) (r) (s) (v) (z)
	Dica e orientação de saúde	“Combinação de vitaminas e minerais que ajuda a suprir parte das necessidades nutricionais do seu filho”; Incentivo da atividade física; Discurso sobre alimentação saudável; Incentivo da atividade física; “O que é Soy force?”; “Comece o dia com sabor e energia!!! Choco ball ajuda a repor rapidamente a sua energia para você poder gastá-la por todo o dia. Inclua em sua alimentação diária, todas as classes de alimentos: frutas, verduras, laticínios e cereais. Estenda a mão e pegue o seu!!!”; “Não adicione sal”; “Frutas são fontes naturais de vitaminas”; “Tenha uma vida mais saudável”; “Alimente-se bem, viva bem!”
	12	(b) (f) (g) (i) (j) (m) (n) (p) (q) (r) (s) (u)
Apelo à saúde (6)	Conteúdo/ ingrediente	“Arroz+Milho+Trigo Levíssima combinação de 3 cereais”; “40% mais cacau, rico em antioxidante”; “Faz bem saber: Os alimentos de origem vegetal possuem lactose?”; “Rico em vitamina C”; “Feito com óleo de girassol”
	6	(a) (g) (i) (m) (r) (v)
	Nutrição	“+ Energia com nutrição”; “Complementa sua alimentação”; “Alimento nutricionalmente completo”; “Energia”; Apresenta um ‘quebra-cabeça’ com vitamina, dizendo que complementa os benefícios do leite
	7	(a) (b) (d) (e) (h) (i) (l)
	Destaque para a formulação do produto	“Nova fórmula”; “Nova receita”; “Fórmula exclusiva”
	5	(a) (b) (e) (l) (v)
	Responsabilidade social	Selo de qualidade – “Pró amendoim ABICAB”
	1	(o)
Propaganda sob propaganda (3)	Disponibilidade de site, redes sociais da marca, QR Code, loja virtual	(a) (b) (c) (d) (e) (f) (g) (h) (i) (j) (k) (l) (m) (n)(o) (p) (q) (r) (s) (t) (u) (v) (x)
	23	
	Estímulo a provar os outros sabores disponíveis e outros produtos da marca	(b) (d) (e) (f) (g) (h) (i) (o) (q) (r) (s) (v) (z)
	13	
	Associação entre duas marcas no mesmo produto	“Tetrapak – Protege o que é bom”; “Marca Coca-Cola”; Logomarca da Danone no rótulo; Logomarca da Pepsico; Logomarca da Arcor
	5	(e) (h) (m) (r) (v)
Apelo a sustentabilidade (2)	Produto reciclável	Selo de embalagem reciclável; “Papel reciclável”; “O papel desta embalagem foi produzido com madeira proveniente de florestas certificadas FSC e outras fontes controladoras”; “Reciclar compromisso de todos”; “Faça coleta seletiva”
	20	(a) (b) (c) (d) (e) (g) (h) (i) (l) (m) (n) (p) (q) (r) (s) (t) (u) (v) (x) (z)
	Preservação do meio ambiente	Mensagem de preservação do meio ambiente; “Embalagem produzida a partir de fontes responsáveis”; “Pratique coleta seletiva”; “Preserve a natureza, recicle a embalagem”; “Mantenha sua cidade limpa”; “A natureza agradece”; “Respeite a vida e a natureza”; “Comprando esta embalagem você ajuda a cuidar das florestas do mundo”; “Preserve o meio ambiente. Economize energia. Recicle materiais”
	15	(a) (b) (c) (e) (g) (h) (i) (l) (m) (n) (q) (r) (s) (t) (v)
Uso da marca/slogan (2)	Grande destaque da marca	(a) (c) (d) (e) (f) (g) (h) (i) (j) (k) (l) (m) (n) (o) (p) (q) (r) (s) (t) (u) (v) (z)
	22	
	Nome da marca ou produto associado a slogan ou texto	“Com Neston tudo fica + gostoso, fica surreal”; “Quem bebe Grapette repete”; “Sustagen + mamãe = aliados por uma nutrição mais completa”; “Nestlé faz bem”; “Comece o dia com actimel”; “Por que as tortinhas Adria são tão gostosas? É simples!..”; “Dá gosto de ter Arisco em casa”; “Qualidade Santa Helena – Irresistível é viver bem”; “Salgadinho deliciosamente divertido da Yoki”; “Com pipoca Yoki tudo que é bom fica mais! o cinema é mais cinema, o futebol é mais futebol, a festa é mais festa”
	14	(b) (c) (d) (e) (f) (h) (i) (l) (m) (n) (o) (u) (v) (x)
Participação de celebridades e personagem (2)	Personagem	Personagem próprio da marca; Personagem da atualidade; Animais e desenhos
	18	(a) (b) (d) (e) (f) (g) (h) (i) (j) (l) (l) (m) (n) (p) (r) (s) (u) (v)
	Personagem humanizados	Presença de desenhos humanizados; Ser humano animado; Alimento humanizado
	6	(b) (c) (e) (h) (i) (j)
Oferta de brinde (2)	Entretenimento infantil	Presença de jogo; Estímulo a acessar o site para jogar jogos disponíveis; Estímulo a entrar no site para diversão; Presença de história em quadrinho; Presença de desafios
	5	(b) (e) (g) (i) (n)
	Brinde e promoção	Brinde; “Promoção – Achou Ganhou”
	5	(c) (g) (h) (i) (s)
Preço promocional (1)	Maior economia	“Mais economia”; “Grátis 100g”; “50% a mais”; “20% do produto grátis”; Sugestão de preço de venda; “Tamanho econômico”; “+ Biscoito + Economia”
	6	(c) (d) (e) (g) (i) (v)

Legenda dos grupos de alimentos: (a) Produtos de carne reconstituída; (b) Farinhas; (c) Refrigerantes; (d) Suplementos e complementos alimentares; (e) Lácteos; (f) Macarrões instantâneos; (g) Pães; (h) Biscoitos doces; (i) Doces, balas e guloseimas; (j) Margarinas; (k) Bolos prontos; (l) Achocolatados em pó e similares; (m) Bebidas de soja; (n) Cereais matinais; (o) Amendoins; (p) Temperos; (q) Sucos concentrados e xaropes; (r) Sucos de caixa; (s) Sucos em pó; (t) Refrescos de guaraná; (u) Papas infantis industrializadas; (v) Biscoitos salgados; (x) Pipocas de micro-ondas; e (z) Farofas prontas.

O recurso “produto” (que abarca presença de imagem do produto, entre outros), referente à ECM “estímulo aos sentidos”, foi utilizado nos rótulos dos 24 grupos de alimentos. O recurso “destaque a aspectos da composição nutricional do produto”, referente à ECM “apelo à saúde”, e o recurso “disponibilização de site, redes sociais da marca, QR code e loja virtual”, referente à ECM “propaganda sob propaganda” foram utilizados nos rótulos de 23 dos 24 grupos de alimentos. Também foram largamente utilizados o recurso “grande destaque da marca” referente à ECM “uso da marca/slogan” (em 22 grupos de alimentos); o recurso “cores” na ECM “estímulo aos sentidos” (em 22 grupos); e o recurso “produto reciclável” referente à ECM “apelo à sustentabilidade” (em 20 grupos).

## DISCUSSÃO

Os AUP analisados possuíam em média 7,2 ECM por rótulo, variando de uma a 19 estratégias, sendo mais recorrentes as de “estímulo aos sentidos” e “apelo à saúde”. Em estudo que analisou 93 rótulos de produtos e avaliou a qualidade de alimentos industrializados comercializados em supermercados da zona sul do Rio de Janeiro, observou-se que até oito ECM foram identificadas em produtos destinados ao público infantil^21^. A presença dessas estratégias nesses rótulos pode acarretar a fidelização à marca ou ao produto^22^ e encoraja crianças a persuadir seus pais a adquiri-lo^23^.

Com exceção do grupo refresco de guaraná, todos os grupos de bebidas apresentaram médias de ECM igual ou superior a 7,4. Outros estudos com metodologia semelhante à deste, realizados com alimentos dirigidos ao público infantil, obtiveram média de 2,4 ECM por rótulo para bebidas gaseificadas ou sucos artificiais em 2012 e média de 4,4 ECM por rótulo para suco, néctares e refrescos de frutas em 2013^21,24^.

A maior recorrência da ECM “estímulo aos sentidos” corrobora o resultado de outro estudo no qual um terço das embalagens continha imagens ou ilustrações de alimento*s in natura* ou do principal ingrediente utilizado^21^. Em produtos voltados para o público infantil comercializados no Uruguai, esta estratégia foi mais recorrente no grupo de biscoitos e doces^20^.

No presente estudo, observou-se que o uso de cores foi recorrente. Tendo em vista que as cores possuem forte apelo visual, trazendo consigo mensagens subliminares e sensações, os profissionais de marketing e propaganda as utilizam nas embalagens para destacar o produto e atrair seu público-alvo. As crianças geralmente são atraídas por cores como amarelo, vermelho, azul e verde. Estas cores têm influência no estímulo, atenção e motivação para a compra e, por outro lado, o azul e o verde oferecem sensação de calma e repouso^25^. No Uruguai, o uso de cores vivas em embalagens de alimentos voltados para crianças é uma estratégia frequentemente utilizada em rótulos de doces e chocolates^20^. Em estudo sobre avaliação da memória visual de embalagens de biscoitos doces e salgadinhos de pacote, as cores mais lembradas pelas crianças em seus desenhos foram vermelho, azul, amarelo e marrom^26^.

Outro aspecto que precisa ser considerado é a presença de alegações nutricionais em embalagens de AUP, categorizadas como um dos tipos de recursos comunicacionais da ECM “apelo à saúde”. Esta foi a segunda ECM mais recorrente, o que converge para os resultados de um estudo que apontou que temas relacionados à saúde, como enriquecimento de nutrientes, relação do consumo com boa saúde e crescimento e completude nutricional, atingiram alta frequência de estratégias persuasivas em rótulos. O uso de alegações sobre vitaminas e minerais adicionados foi recorrente nos produtos alimentícios destinados ao público infantil (laticínios, biscoitos, produtos de pastelaria, cereais matinais, comida instantânea e refrigerantes e sucos)^20^.

O destaque de uma ou mais alegações nutricionais no rótulo do alimento pode transmitir a ideia enganosa de alimento saudável, produzindo uma percepção positiva entre os consumidores^27^. Estudo realizado no Brasil mostrou que alegações nutricionais em embalagens de AUP destinados ao público infantil influenciaram a percepção de crianças entre 8 e 10 anos sobre a qualidade do produto como um todo^28^.

A adoção de dietas sustentáveis vem sendo estimulada por especialistas e instituições de todo o mundo, como a Organização das Nações Unidas para a Alimentação e a Agricultura (FAO)^29^. Nesse sentido, o “apelo à sustentabilidade” aparece entre as ECM praticadas pelas indústrias de alimentos, conforme se viu no presente estudo. Entretanto, na maioria dessas mensagens, o foco não estava no processo produtivo dos AUP, mas sim na responsabilização do consumidor em relação à sustentabilidade ambiental, reforçando mensagens sobre o descarte em lixo próprio, por exemplo.

O destaque da marca e slogan foi a terceira ECM mais aplicada nos rótulos. A marca é um importante elemento da propaganda, uma vez que influencia favoravelmente a decisão de compra pelo consumidor, além de identificar o produto e diferenciá-lo dos concorrentes^26^. Ao promover a marca, todos os produtos que a empresa comercializa são favorecidos^30^. Em outro estudo sobre a mesma temática, realizado com pais de crianças com idade entre 2 e 12 anos, o reconhecimento da marca e do fabricante foi o segundo principal fator de decisão de compra pelos pais^31^.

A compreensão sobre como diferentes ECM alcançam adultos e/ou crianças ainda requer consolidação^32^. Uma revisão de escopo identificou estudos que rastrearam tipos de estratégias de comunicação ou que mediram sua eficácia e concluiu que há necessidade de pesquisas futuras sobre o poder de persuasão dessas estratégias a depender do seu tipo e da idade das crianças^14^. Um estudo experimental com pares de crianças e responsáveis revelou que a publicidade de produtos não saudáveis promoveu percepções favoráveis de produtos e aumentou as preferências dos responsáveis pelos produtos anunciados. Também observou que intervenções de contrapropaganda podem reforçar a resistência dos responsáveis à publicidade persuasiva destes produtos e capacitá-los a avaliar com mais precisão aqueles não saudáveis^33^. Considera-se que a regulação de rotulagem frontal poderia constituir mais um elemento formativo das famílias e ser uma ferramenta de educação e saúde^34^.

A oferta de brindes foi uma das ECM menos frequentes nesse estudo, diferentemente do observado na literatura, que aponta frequência de 3,6% a 10% de presença de oferta de brindes, jogos ou brincadeiras e sorteio de prêmios^21,24^. Nossos resultados podem ter subestimado a oferta de brindes porque a alusão ao brinde pode ser feita via TV ou internet ou, ainda, ser uma campanha sazonal e o momento da pesquisa não coincidir com ela.

Uma hipótese alternativa seria o estudo ter captado uma tendência de redução na oferta de brindes atrelados aos produtos de consumo infantil. Uma potencial explicação seria a implementação da Resolução n° 163, de 13 de março de 2014, do Conselho Nacional dos Direitos da Criança e do Adolescente (Conanda), que dispõe sobre a abusividade da publicidade e comunicação mercadológica destinadas à criança e ao adolescente, descrevendo, no Art. 2°, como abusivo, o uso de promoções de distribuição de prêmios e brindes colecionáveis ou com apelo infantil^35^. A publicidade infantil abusiva e enganosa infringe a Constituição Federal, o Estatuto da Criança e do Adolescente e o Código de Defesa do Consumidor, uma vez que esses dispositivos legais visam proteger as crianças, consideradas vulneráveis, em função de sua incapacidade de averiguar os interesses comerciais e persuasivos das peças publicitárias^36^.

A regulação da rotulagem nutricional frontal de advertência é uma das medidas prioritárias da agenda regulatória voltada à prevenção e ao controle das doenças crônicas não transmissíveis^37^. Além de garantir à população informação mais clara e objetiva sobre a real composição nutricional dos AUP, os dispositivos legais também podem prever mecanismos para limitar a presença de ECM em rótulos e até a oferta desses alimentos em ambientes alimentares, como ocorreu no Chile^38^. Já no Brasil, a norma técnica aprovada pela Agência Nacional de Vigilância Sanitária (Anvisa) para adequação da rotulagem frontal dos alimentos processados e ultraprocessados (Resolução da Diretoria Colegiada – RDC 429/2020) ficou restrita à informação sobre composição nutricional e não avançou na regulação de ECM em rótulos^39^.

As principais limitações do estudo foram: a impossibilidade de análise de 15% dos 459 produtos referidos pelos participantes do inquérito que originou o presente estudo e a análise parcial de 20,3% dos 390 rótulos incluídos no estudo. A análise de um menor elenco de produtos e de parte das faces dos rótulos de alguns daqueles analisados pode levar a erros (para mais ou para menos) nas estimativas de frequência percentual e de média das ECM examinadas. Outro aspecto que merece comentário é o fato de que, no inquérito, os R24h foram aplicados entre terça e sexta-feira, não cobrindo, portanto, o consumo alimentar em fins de semana. Isso pode ter subestimado o elenco de AUP consumidos pelas crianças estudadas, supondo-se que a presença de AUP seja maior em dias de fim de semana.

Por outro lado, a análise de rótulos de AUP efetivamente consumidos por uma amostra probabilística de crianças usuárias do SUS constitui-se como uma fortaleza desse estudo. Comparada à abordagem mais recorrente na literatura (análise de produtos disponíveis em estabelecimentos comerciais)^14^, esta escolha tem como vantagem o conhecimento dos produtos efetivamente consumidos pelas crianças, independentemente de terem sido dirigidos a elas. Isso pode ajudar a subsidiar políticas públicas tanto de aconselhamento na atenção primária à saúde quanto de regulação de rótulos de alimentos.

## CONCLUSÃO

No presente estudo foram identificadas ECM em todos os rótulos dos AUP e papas infantis industrializadas consumidos por crianças. É fundamental adequar e aperfeiçoar a rotulagem de alimentos destinados tanto ao público infantil quanto ao público em geral, com base em medidas regulatórias que coíbam práticas enganosas e abusivas. Isso porque os diversos tipos de ECM empregadas nos rótulos, principalmente aqueles que instigam os sentidos, exaltam aspectos relacionados à saúde e colocam a marca em destaque, podem gerar o consumo exacerbado de alimentos de baixo teor nutricional, favorecendo o aumento da obesidade infantil e impactos negativos na saúde. O reconhecimento do risco oferecido à saúde e nutrição infantil pelas ECM reafirma a necessidade de medidas regulatórias rigorosas que protejam o consumidor da massiva exposição às ECM em rótulos de alimentos.

São também fundamentais políticas públicas que estimulem a difusão de informações confiáveis, pautadas nos guias alimentares, para toda a população e em diferentes mídias. O uso dos guias alimentares por profissionais de saúde, educação, assistência social e outros pode qualificar ações de educação alimentar e nutricional que abordam a leitura e compreensão de rótulos, ampliar o senso crítico de famílias e promover uma alimentação mais adequada e saudável.
